# Re-Inspection of Small RNA Sequence Datasets Reveals Several Novel Human miRNA Genes

**DOI:** 10.1371/journal.pone.0010961

**Published:** 2010-06-04

**Authors:** Thomas Birkballe Hansen, Jesper Bertram Bramsen, Jørgen Kjems

**Affiliations:** Department of Molecular Biology, Aarhus University, Aarhus, Denmark; New England Biolabs, Inc, United States of America

## Abstract

**Background:**

miRNAs are key players in gene expression regulation. To fully understand the complex nature of cellular differentiation or initiation and progression of disease, it is important to assess the expression patterns of as many miRNAs as possible. Thereby, identifying novel miRNAs is an essential prerequisite to make possible a comprehensive and coherent understanding of cellular biology.

**Methodology/Principal Findings:**

Based on two extensive, but previously published, small RNA sequence datasets from human embryonic stem cells and human embroid bodies, respectively [1], we identified 112 novel miRNA-like structures and were able to validate miRNA processing in 12 out of 17 investigated cases. Several miRNA candidates were furthermore substantiated by including additional available small RNA datasets, thereby demonstrating the power of combining datasets to identify miRNAs that otherwise may be assigned as experimental noise.

**Conclusions/Significance:**

Our analysis highlights that existing datasets are not yet exhaustedly studied and continuous re-analysis of the available data is important to uncover all features of small RNA sequencing.

## Introduction

miRNAs are small ∼22 nt non-coding RNA sequences. The miRNA is produced by the cellular RNAi machinery from large hairpin structured transcript (pri-miRNA) into the mature form (miRNA) in a two-step process having a precursor miRNA (pre-miRNA) intermediate (reviewed in [2]). The primary function of miRNAs is currently seen as targeting partly complementary sequences in mRNA 3′ UTRs, hereby modulating mRNA stability and affecting translational efficiencies (reviewed in [3]).

To date, 721 human miRNAs (miRBase 14.0 [4]) have been annotated, a number that is continuously increasing. Since the discovery of miRNAs, several attempts to predict and discover miRNA genes have been made either using a comparative phylogenetic approach [5], [6] or a non-comparative, support vector machine based approach [7]–[9]. With the emergence of high-throughput sequencing techniques the algorithms are backed up by experimentally based datasets which greatly enhance the predictive power [10]. However, annotating novel miRNAs solely based on sequencing and bioinformatics should be undertaken only cautiously as the high sensitivity of sequencing techniques are bound to collide with the enormous quantities of pseudo hairpins found in the human genome [11]. Considering the complex combinatorial regulatory functions ascribed to miRNAs today, it is of great importance to find and validate all genomic miRNAs in humans and other organisms to uncover the complexity of gene regulation during cellular differentiation, homeostasis and disease [12]. Furthermore, the identification of all miRNAs would greatly improve the quality of available training-sets for the bioinformatical predictions of additional miRNA structures and miRNA targets in contrast to falsely annotated miRNAs that would seriously hamper such analysis. By re-inspection of published small RNA sequence datasets [1], [13]–[18], we here put forth an extensive list of yet 112 un-annotated miRNA candidates with 12 of 17 putative miRNAs being validated by northern blotting giving a preliminary 70% success rate of our prediction.

## Results and Discussion

Datasets obtained from high throughput sequencing of human embryonic stems cells (hESC) and human embroid bodies (hEB) sequences [1] were analysed for known and novel miRNA structures (see Materials and Methods section for a detailed description); by genomic BLAT, secondary structure prediction including adjacent genomic sequences and finally assessing the similitude towards well-known miRNA structures. The datasets combined contained approximately 80% of all annotated miRNAs and generated an extensive list of yet un-annotated miRNA-like structures (Table S1, Figure S2 and Table S2). To evaluate the method of prediction, a subset of miRNA candidates were subjected to experimental validation: A total of 17 candidates were randomly picked, inserted along with approximately 250 nt flanking genomic sequence on either side into an intron of an eGFP expression plasmid (pJEBB, Figure S3), and overexpressed individually in HEK293 cells. Including the flanking sequences allows expressing the candidates as putative pri-miRNA mimics thus requiring sequential Microprocessor and Dicer cleavage for maturation. Probing for the mature miRNA (band of ∼22 nt) on a northern blot indicate whether the cellular miRNA-processing machinery recognizes the sequence and structure as a miRNA or not. Whereas positive northern signal is a very strong evidence of a bone fide miRNA, it should be noted that failure to detect any given mature miRNA band does not necessarily infer false prediction, due to the suboptimal sensitivity of northern blotting (i.e. using DNA probes). As seen in Fig S1 and Table S1, 11 out of 17 miRNA candidates were clearly positive on northern blots, suggesting that at least 11 novel miRNAs were identified; however, after the initial screen candidate 112 (miR-1911) has been included in the miRBase in addition to one of our un-validated candidates (candidate 111, miR-1912). In case of candidate 39 (Figure S2), no clear mature product was observed; instead a distinct band matching the approximate size of the pre-miRNA appeared. We speculate that the missing mature band is either due to rapid turnover of the processed miRNA [19], an example of post-transcriptional regulation of miRNA processing [20]–[24] or perhaps not a suitable Dicer substrate as previously observed for hairpin structures in the 5′UTR of DGCR8 mRNA [25]. The remaining 5 of 17 failed to be detected suggesting that these particular miRNA candidates are processed inefficiently in HEK293 cells, undetectable due to technical limitations or simply not valid miRNAs.

Our results suggest that the combination of high-throughput sequencing datasets allows the identification of novel miRNAs that in the individual datasets are expressed at close to noise levels. In this regard, miRNAs are highly expressed at only very limited temporospatial windows during e.g. embryonic development and cellular differentiation [26], [27] and a low representation in the available sequencing sets is thereby not reflective of poor biological significance of the particular miRNA. Therefore, we propose that available miRNA sequencing datasets should be combined to raise the signal-to-noise ratio in miRNA identification based on small-RNA sequencing. Notably, most miRNAs predicted and verified here are very poorly conserved (Table S1), which is expected taken the rapid evolution of miRNAs in higher eukaryotes into account [28]. This is also an outcome of the fact that most miRNA prediction tools take phylogenetic conservation as a strong validation parameter, thus generating a bias towards the non-conserved in the pool of yet to be discovered miRNAs. Consequently, many valid miRNAs will remain undisclosed during data analysis unless the requirement for cross species conservation is relaxed.

We find that 52 out of the 112 suggested miRNAs have reads positioned both on the 3 p and 5 p arm which is normally seen as strong bioinformatic evidence. However, in two cases (candidate 41 and candidate 101) we failed to pick up a mature miRNA product despite the rather convincing secondary RNA structure coupled with 5 p/3 p reads. Unless this is an example of northern blotting not having the required sensitivity, in which case other means should be pursued in order to validate the putative miRNA, eg. luciferase reporter assay or splinted ligation [29], or caused by unforeseen elements in the experimental vector or cell-line abolishing the processing of these particular miRNAs, this could indicate that even strong evidence based on high throughput sequencing seems as inadequate proof for the existence of bona fide miRNAs. Furthermore, annotated miRNAs like miR-608 [30] and miR-623 [30], which are not picked up in all the datasets here investigated and unsuitable to experimental validation in our hands, are probably two examples out of several falsely annotated miRNAs in the miRBase. Thus, we strongly advise that miRNAs should preferentially be validated experimentally, e.g. by overexpression and northern blotting, before submission to the miRBase, and that successful validation should be a necessary criteria in the conventions for miRNA annotation [31].

## Materials and Methods

### Dataset analysis

Datasets were obtained from high throughput sequencing of human embryonic stems cells (hESC) [1] and human embroid bodies (hEB) [1]. Sequences more than 17 nucleotides in length and with at least two reads (24699 and 18347 sequences for hESC and hEB, respectively) were BLATed against the human genome (UCSC Genome Browser, hg18). Sequences with more than two genomic hits were discarded to avoid multi-mapping sequences and to limit the dataset (4961 and 3166, respectively) leaving 19738 and 15181 sequences with 22548 and 17306 genomic hits, respectively. Hits mapping to annotated regions (UCSC Tables; RNA genes and sno/miRNA) were then eliminated (10561 and 8445, respectively) and RNA secondary structure prediction (MultiRNAfold version 1.1, [32]) were conducted on the remaining hits (11987 and 8861, respectively) including 70 nt of flanking genomic sequences. Here, predicted RNA structures having at least a 23 bp stem with at most 5 unpaired nucleotides on each arm were manually inspected, and miRNA-like structures with sequence reads positioned on the hairpin stem were included in the candidate list. Subsequently, all miRNA candidates were cross-examined with additional small RNA sequence datasets [13]–[18] to further strengthen the validity of the proposed miRNAs. Conservation was determined using the 28-way alignment score provided by UCSC Genome Browser.

Structures, as seen in Figure S4, are all folded using the MultiRNAFold package and visualized with RNA Folder (http://www.rnai.dk/index.php/software.html).

### Cloning, expression and northern blot

Selected miRNAs were PCR amplified with primers listed in Table S3, digested with NotI and SalI (NotI and XhoI in case of candidate 48) and ligated into intron of eGFP expressing plasmid (pJEBB, Figure S3, unpublished). Plasmids were transiently transfected into HEK293 (Flp-InTM T-RexTM 293 Cell Line, Invitrogen, Carlsbad CA) using calcium phosphate protocol. After 48 hrs, RNA was harvested using TriZol® (Invitrogen) adhering to manufacturers protocol. Finally, 30 ug RNA was loaded onto 12% PAGE, transferred to Amersham hybond^TM^-N+ membrane (GE Healthcare, Fairfield CT) and hybridized with ^32^P end-labelled DNA probes (Table S3) in church buffer (0.5 M NaPO_4_, 7% SDS, 1 mM EDTA, 1% BSA, pH 7.5) at 37°C and washed in SSC buffer (2xSSC, 0.1% SDS) at room temperature. The membranes were exposed on phosphorimager screens and analysed using Bio-Rad Quantity One® software (Bio Rad, Hercules CA).

## Supporting Information

Figure S1 Validation by northern blotting. a–k) Northern blotting with RNA from HEK293 cells transiently transfected with a miRNA expressing plasmid or an empty vector. Membranes were probed with DNA oligo complementary to the putative mature sequence that based on sequencing datasets were expectedly produced from each miRNA (upper panel) or probed against endogenously expressed miR-15b (lower panel).
(0.13 MB PDF)

Figure S2 Candidate 39. Northern blotting with RNA from HEK293 cells transiently transfected with candidate 39 or an empty vector. Membranes were probed with either DNA oligoes complementary to the putative mature sequence (top panel), the loop sequence (middle panel) or the endogenously expressed miR-15b (lower panel). The mature band arrow in the upper panel (mature*) points to the expected migration of the mature strand, however, no detectable band appears.
(0.05 MB PDF)

Figure S3 Schematic representation of pJEBB; vector used for miRNA overexpression. pJEBB is composed of a CMV promoter, eGFP ORF, an intronic pri-miRNA expression cassette flanked by splice-donor (SD) and splice-acceptor (SA) sequences, and a b-globin poly(A) (pA) termination signal.
(0.00 MB PDF)

Figure S4 Figure S4, Secondary structure of miRNA candidates. Using RNA Folder interface and the MultiRNAFold package all miRNA candidates are structurally presented including ∼20 flanking nucleotides on each side. Mature sequences, as obtained from high throughput datasets, are depicted in red.
(0.41 MB PDF)

Table S1 Novel miRNA candidates, Compiled list of all the putative miRNA candidates. In all cases, mature sequence, chromosomal positions, genomic hit count and available reads from each dataset examined have been included. Successful or failed detection of mature miRNA is marked by green or red, respectively. (* Validation is solely based on pre-miRNA detection; ** After the validation this miRNA was by others submitted to the miRBase).
(0.06 MB XLS)

Table S2 miRNA sequences and reads. All miRNA candidates are presented with primary sequence (including 20 flanking nucleotides, cf. Figure S4), dot-bracket structure obtained from MultiRNAFold algorithm and aligned reads from each individual dataset (clustered reads from each dataset are presented with sequence and read-count in square brackets).
(0.24 MB DOC)

Table S3 List of primers and probes.
(0.03 MB XLS)
