# Supplementary figures and images for: Re-Inspection of Small RNA Sequence Datasets Reveals Several Novel Human miRNA Genes
Source: PLoS One. 2010 Jun 4;5(6):e10961. doi: 10.1371/journal.pone.0010961 (PMC2881037; doi:10.1371/journal.pone.0010961)

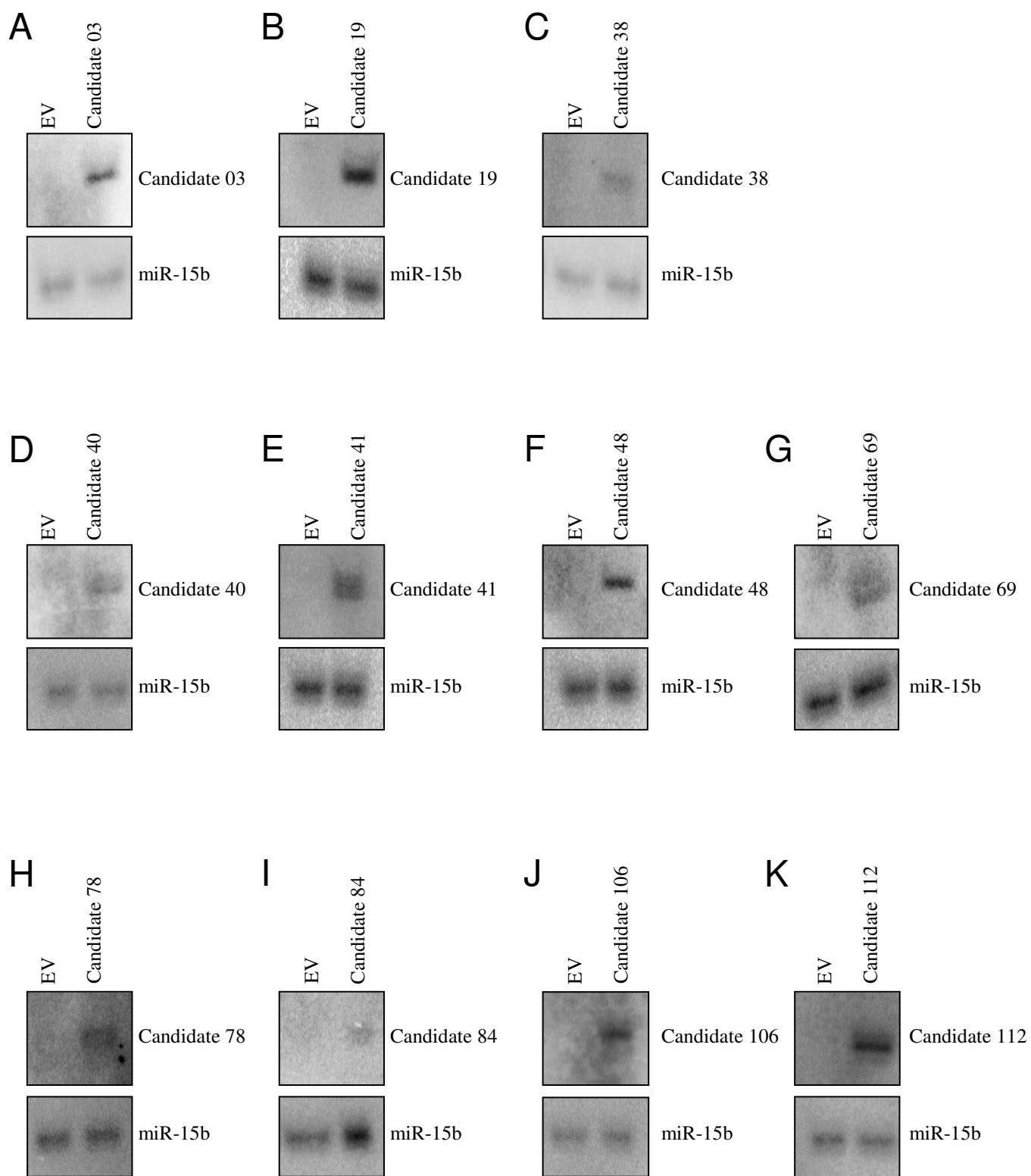

Supplement: Figure S1 — Validation by northern blotting. a–k) Northern blotting with RNA from HEK293 cells transiently transfected with a miRNA expressing plasmid or an empty vector. Membranes were probed with DNA oligo complementary to the putative mature sequence that based on sequencing datasets were expectedly produced from each miRNA (upper panel) or probed against endogenously expressed miR-15b (lower panel). (0.13 MB PDF) [file pone.0010961.s001.pdf]

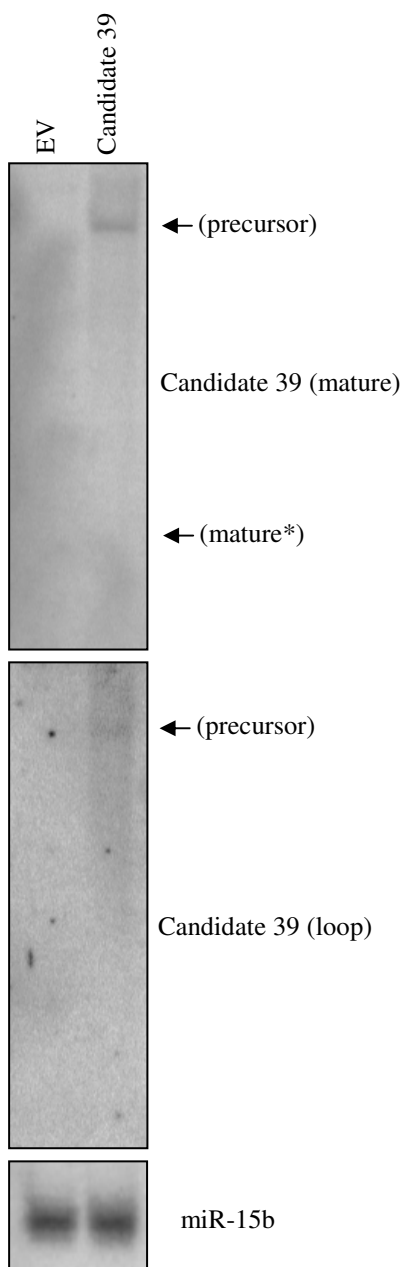

Supplement: Figure S2 — Candidate 39. Northern blotting with RNA from HEK293 cells transiently transfected with candidate 39 or an empty vector. Membranes were probed with either DNA oligoes complementary to the putative mature sequence (top panel), the loop sequence (middle panel) or the endogenously expressed miR-15b (lower panel). The mature band arrow in the upper panel (mature*) points to the expected migration of the mature strand, however, no detectable band appears. (0.05 MB PDF) [file pone.0010961.s002.pdf]

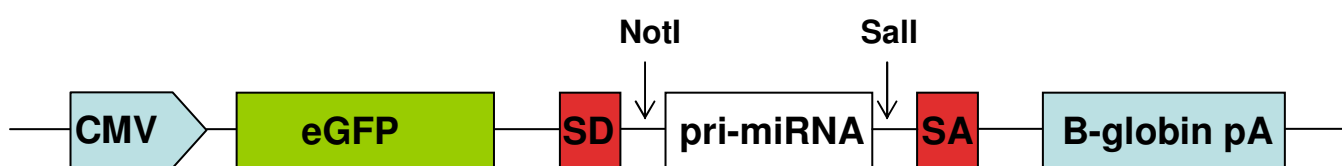

Supplement: Figure S3 — Schematic representation of pJEBB; vector used for miRNA overexpression. pJEBB is composed of a CMV promoter, eGFP ORF, an intronic pri-miRNA expression cassette flanked by splice-donor (SD) and splice-acceptor (SA) sequences, and a b-globin poly(A) (pA) termination signal. (0.00 MB PDF) [file pone.0010961.s003.pdf]

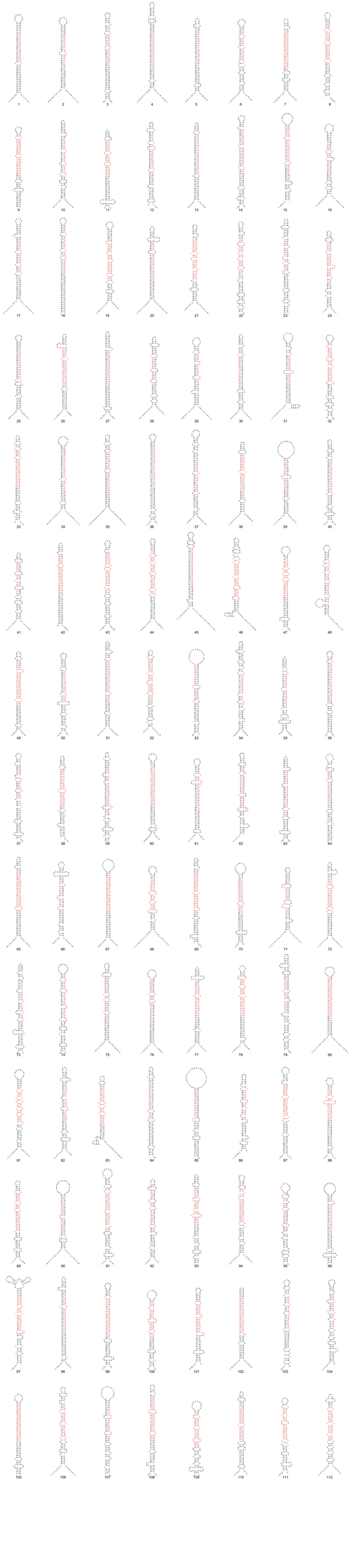

Supplement: Figure S4 — , Secondary structure of miRNA candidates. Using RNA Folder interface and the MultiRNAFold package all miRNA candidates are structurally presented including ∼20 flanking nucleotides on each side. Mature sequences, as obtained from high throughput datasets, are depicted in red. (0.41 MB PDF) [file pone.0010961.s004.pdf]
