# Supplementary material for: Re-Inspection of Small RNA Sequence Datasets Reveals Several Novel Human miRNA Genes
Source: PLoS One. 2010 Jun 4;5(6):e10961. doi: 10.1371/journal.pone.0010961 (PMC2881037; doi:10.1371/journal.pone.0010961)
Supplement: Table S2 — miRNA sequences and reads. All miRNA candidates are presented with primary sequence (including 20 flanking nucleotides, cf. Figure S4), dot-bracket structure obtained from MultiRNAFold algorithm and aligned reads from each individual dataset (clustered reads from each dataset are presented with sequence and read-count in square brackets). (0.24 MB DOC) [file pone.0010961.s006.doc]

1 AAUGUGUGAGCACCAGGGGUACCUCUCUACUGACUUGCAACAUACAUUUGUCUUGGUGUGUUGCAAGUCGGUGGAGACGUACCCUUGGUGCUCAACCCCU

......((((((((((((((((.((((((((((((((((((((((..........)))))))))))))))))))))).))))))))))))))))......

Morin (hEB) UGUUGCAAGUCGGUGGAGACG[1]

Morin (hESC) UGUUGCAAGUCGGUGGAGACGUA[1]

Morin (hESC) UGUUGCAAGUCGGUGGAGACGU[2]

Morin (hESC) UGUUGCAAGUCGGUGGAG[2]

2 UGUGCUAAGUGCUUUACAUGAAUGGUCCCAUUGAAUCCCAACAGCUUUGCGAAGUGUUGUUGGGAUUCAGCAGGACCAUUCGUGUAAAGUAAUAUUCCCA

.........(((((((((((((((((((..(((((((((((((((..........)))))))))))))))..))))))))))))))))))).........

ENCODE (GSE14362) UGUUGGGAUUCAGCAGGACCAU[1]

Morin (hEB) UUGGGAUUCAGCAGGACCAUU[1]

Morin (hESC) UUGGGAUUCAGCAGGACCAU[1]

Morin (hESC) UGUUGGGAUUCAGCAGGACCA[1]

Morin (hESC) UGGGAUUCAGCAGGACCAUU[2]

Taft (FANTOM4) UGUUGGGAUUCAGCAGGACCA[1]

Taft (FANTOM4) UGUUGGGAUUCAGCAGGACCAU[2]

Taft (FANTOM4) UGUUGGGAUUCAGCAGGACCAUU[1]

3 UCUACAAGCAGAUACAAGGAUGCCCUUGUACACAACACACGUGCUGCUUGUAUAGACAUGAGUGUUGUCUACGAGGGCAUCCUUGUGUCUGUGUGUGUG

..((((.(((((((((((((((((((((((.(((((((.(((((((......))).)))).))))))).))))))))))))))))))))))).))))..

ENCODE (GSE14362) UGAGUGUUGUCUACGAGGG[1]

ENCODE (GSE14362) UGAGUGUUGUCUACGAGGGC[2]

ENCODE (GSE14362) UGAGUGUUGUCUACGAGGGCAU[4]

Morin (hEB) UGAGUGUUGUCUACGAGGGCAU[10]

Morin (hEB) UGAGUGUUGUCUACGAGGGC[2]

Morin (hESC) UGAGUGUUGUCUACGAGGGCAU[2]

Morin (hESC) UGAGUGUUGUCUACGAGGGCA[1]

Morin (hESC) UGAGUGUUGUCUACGAGG[1]

4 AUGUCCUUUUAACAUCUUUUCCAUCAUAAUUCUCAUAGUAAUAAUAGUAAUGUUAAUAUAAUAAUAAUGUUAUUAUUACUGUGAGAAUUAUGAUGGAAAAGAAGUUAUUACUAUGA

.........((((.(((((((((((((((((((((((((((((((((((.(((((......))))).))))))))))))))))))))))))))))))))))).)))).........

Morin (hEB) UUACUGUGAGAAUUAUGAUGG[1]

Morin (hESC) UUACUGUGAGAAUUAUGAUGG[2]

Morin (hESC) AUUACUGUGAGAAUUAUGAUGG[1]

5 UUCAUCAAGCACUUUAUUGAGUCCCUACUAUGUUCCAGGCACUGGGUAUCGUAGGUGCCUGGAACAUAGUAGGGACUCAAUAAAGGAGCUAUGCUU

.......(((.((((((((((((((((((((((((((((((((..(.....).))))))))))))))))))))))))))))))))..)))......

ENCODE (GSE14362) UGCCUGGAACAUAGUAGGGACU[1]

Morin (hEB) UGCCUGGAACAUAGUAGGGACU[5]

Morin (hESC) UGCCUGGAACAUAGUAGGGACU[6]

Morin (hESC) UGCCUGGAACAUAGUAGGGA[1]

6 UCGAAUUUCCCUAAAGGGCCAGACACUAUACGAGUCAUAUAAGGGAAGGCAUUAUAGGACUCAUAUAGUGCCAGGUGUUUUGUGGGACACUAU

.......(((((((((.(((.(.(((((((.(((((.(((((.........))))).))))).))))))).).))).))))).))))......

Morin (hEB) AUAGGACUCAUAUAGUGCCAGG[2]

Morin (hEB) UAGGACUCAUAUAGUGCC[1]

Morin (hEB) AUAGGACUCAUAUAGUGCCAG[1]

Morin (hESC) AUAGGACUCAUAUAGUGCCAG[3]

7 AUUGCUUCUUAAAUGGUUAUGUCCUUUGCCUAUUCUAUUUAAGACACCCUGUACCUUAAAUAGAGUAGGCAAAGGACAGAAACAUUUUAAGUUCUCU

.......((((((..(((.((((((((((((((((((((((((((.....))..))))))))))))))))))))))))..)))..))))))......

ENCODE (GSE14362) UAAAUAGAGUAGGCAAAGGA[1]

Morin (hEB) UAAAUAGAGUAGGCAAAGGACA[1]

Morin (hEB) UCCUUUGCCUAUUCUAUUUAAG[1]

Morin (hESC) UAAAUAGAGUAGGCAAAG[1]

Morin (hESC) UAAAUAGAGUAGGCAAAGGACAG[1]

Morin (hESC) UAAAUAGAGUAGGCAAAGGAC[3]

Morin (hESC) UAAAUAGAGUAGGCAAAGGACA[10]

8 GCUUGCAUAAAAUUGCAGUGCCUAAUUUGAACACCUUCGGUAUUCAUCAAAAAUACCAAAGGUGCUCAAAUUAGACAUUGCAUUUUUUGCGCAUGU

((..(((.((((.(((((((.(((((((((.((((((.((((((.......)))))).)))))).))))))))).))))))).)))))))))....

Morin (hEB) AAAGGUGCUCAAAUUAGACAUU[1]

Morin (hEB) CCUAAUUUGAACACCUUC[1]

Morin (hEB) CCUAAUUUGAACACCUUCGGUA[1]

Morin (hESC) CCUAAUUUGAACACCUUCGGUAU[1]

Morin (hESC) CCUAAUUUGAACACCUUCGGUA[4]

9 GGGCUGACCCCUAGGGUCAGGUGAGGCCCUUGGGGCACAGUGGUGCCAUCUCCCCUGUGCUCCCAGGGCCUCGCCUGUCCCUUGAGGUCGGCCC

(((((((((.(.((((.(((((((((((((.(((((((((.((........)).))))))))).))))))))))))).)))).).)))))))))

Morin (hESC) CAGGGCCUCGCCUGUCCCUU[1]

Morin (hESC) CAGGGCCUCGCCUGUCCCUUG[1]

Morin (hESC) CCUGUGCUCCCAGGGCCUCGC[2]

Taft (FANTOM4) CAGGGCCUCGCCUGUCCCUUGA[1]

Taft (FANTOM4) CCAGGGCCUCGCCUGUCCCUUGA[1]

Taft (FANTOM4) CCUGUGCUCCCAGGGCCUCGC[17]

Taft (FANTOM4) CCUGUGCUCCCAGGGCCUCGCC[1]

Taft (FANTOM4) UGAGGCCCUUGGGGCACAGUGG[1]

10 UCACUCUGCUUCACUGAAGCCUCUUGGGGCUUAUUUAGACAAUGGUUUCAUCAUUUCGUCUGAAUAGAGUCUGAAGAGUCUUUGGUGGAAGUAUGCACU

......((((((((..(((.(((((.((((((((((((((((((((...))))))..)))))))).)))))).))))).)))..)).))))))......

Morin (hEB) UCUGAAUAGAGUCUGAAGAGUC[1]

Morin (hEB) UCUGAAUAGAGUCUGAAGAGUCU[2]

Morin (hESC) UCUGAAUAGAGUCUGAAGAGUC[3]

Morin (hESC) UCUGAAUAGAGUCUGAAGAGU[1]

11 CGUGUCAACUGUUAGGUUGGUGCAAAAGUAAUUGUGGUUUUUGAAAGUAACUUGGCGAAAACGACAAUGACUUUUGCACCAAUCUAAUACAAUCAACA

.((......((((((((((((((((((((.(((((.(((((((.(((...)))..))))))).))))).))))))))))))))))))))......)).

ENCODE (GSE14362) AAAAGUAAUUGUGGUUUUU[2]

ENCODE (GSE14362) AAAAGUAAUUGUGGUUUUUG[5]

ENCODE (GSE14362) AAAGUAAUUGUGGUUUUUG[1]

ENCODE (GSE14362) GUUGGUGCAAAAGUAAUUGUGGUU[1]

Morin (hEB) GAAAACGACAAUGACUUUUGCAC[1]

Morin (hEB) GAAAACGACAAUGACUUUU[1]

Morin (hESC) AAAAGUAAUUGUGGUUUUUGAA[1]

Morin (hESC) CAAAAGUAAUUGUGGUUUUUGAA[1]

Morin (hESC) GAAAACGACAAUGACUUUU[1]

Morin (hESC) GAAAACGACAAUGACUUUUGCA[2]

Taft (FANTOM4) AAAAGUAAUUGUGGUUUUU[1]

Taft (FANTOM4) AAAAGUAAUUGUGGUUUUUG[3]

Taft (FANTOM4) AAAAGUAAUUGUGGUUUUUGA[1]

Taft (FANTOM4) AAAGUAAUUGUGGUUUUUG[1]

Taft (FANTOM4) CAAAAGUAAUUGUGGUUUUUG[5]

Taft (FANTOM4) CAAAAGUAAUUGUGGUUUUUGA[1]

Wyman (GSM379265) CAAAAGUAAUUGUGGUUUU[4]

Wyman (GSM379266) AAAAGUAAUUGUGGUUUUU[1]

Wyman (GSM379267) CAAAAGUAAUUGUGGUUUU[2]

Wyman (GSM379267) AAAAGUAAUUGUGGUUUUU[1]

Wyman (GSM379268) AAAGUAAUUGUGGUUUUUG[1]

Wyman (GSM379268) AAAAGUAAUUGUGGUUUUU[1]

Wyman (GSM379268) CAAAAGUAAUUGUGGUUUU[1]

Wyman (GSM379268) UGGUGCAAAAGUAAUUGUG[1]

12 AAUAAAAGCCUUAUGAUUCUAAUGUCAGAGAUCCUGUCUAUGGGAACAGUGGGCUCCCCAUACACUUGAUCUCUGAUACUAGAAUCAAGGCUUUCGUCAG

.(..(((((((..((((((((.(((((((((((..((.((((((...((....)).)))))).))..))))))))))).)))))))))))))))..)...

Morin (hESC) AUACACUUGAUCUCUGAUACUA[1]

Morin (hESC) CAUACACUUGAUCUCUGAUACU[2]

13 UCUUCGUAAGUUAUAUAUGUCUACUUCUACCUGUGUUAUCAUAAUAAAGGUGUCAUGAUGAUACAGGUGGAGGUAGAAAUAUAUAACUUAUCUCUU

.....(((((((((((((.(((((((((((((((((((((((.(((....))).))))))))))))))))))))))).))))))))))))).....

ENCODE (GSE14362) UGAUGAUACAGGUGGAGGUAG[1]

ENCODE (GSE14362) UGAUGAUACAGGUGGAGGUAGA[1]

Morin (hEB) UGAUGAUACAGGUGGAGGUAG[2]

Morin (hESC) UGAUGAUACAGGUGGAGGUAGA[1]

Morin (hESC) UGAUGAUACAGGUGGAGGUAG[1]

14 GAUGUUCUUUCCCCCAUCCUCCUUACGUCCCACCCCCCACUCCUGUUUCUGGUGAAAUAUUCAAACAGGAGUGGGGGUGGGACAUAAGGAGGAUAGGGAACACAG

..(((...(((((..((((((((((.(((((((((((.(((((((((..(((........))))))))))))))))))))))).)))))))))).))))).))).

Morin (hESC) ACAGGAGUGGGGGUGGGACAU[2]

Morin (hESC) ACAGGAGUGGGGGUGGGAC[2]

Morin (hESC) ACAGGAGUGGGGGUGGGACAUA[2]

Taft (FANTOM4) ACAGGAGUGGGGGUGGGACAU[1]

15 CUGAAGAGUAGAGUGUGUGGUCCCAGUUCAGAAGUGUUCCUGAGUAACUUGUGCUUAUAACUCAGGACACUUCUGAACUUGGACCAUACAGGUCUCCCUGCUU

.....((((((((((((((((((.(((((((((((((.(((((((..............)))))))))))))))))))).))))))))))...)))..)))))

ENCODE (GSE14362) CAGUUCAGAAGUGUUCCUGAG[1]

ENCODE (GSE14362) CAGUUCAGAAGUGUUCCUGAGU[14]

ENCODE (GSE14362) CAGUUCAGAAGUGUUCCUGAGUA[1]

ENCODE (GSE14362) CAGUUCAGAAGUGUUCCUGAGUAA[1]

ENCODE (GSE14362) CCAGUUCAGAAGUGUUCCUGAGU[4]

Morin (hEB) UCAGGACACUUCUGAACUUGGA[2]

Taft (FANTOM4) CAGUUCAGAAGUGUUCCUGAGU[1]

16 UAGUUAGAGGCCCCACGUGGUGAGGAUAUGGCAGGGAAGGGGAGUUUCCCUCUAUUCCCUUCCCCCCAGUAAUCUUCAUCAUGCGGUGUCCCCAGUCCU

........(((.((.((((((((((((.(((..((((((((((...........)))))))))).)))...)))))))))))).)).))).........

ENCODE (GSE14362) UGAGGAUAUGGCAGGGAAG[1]

ENCODE (GSE14362) UGAGGAUAUGGCAGGGAAGGG[2]

Morin (hEB) UGAGGAUAUGGCAGGGAAG[1]

Morin (hEB) UGAGGAUAUGGCAGGGAAGG[1]

Morin (hEB) UGAGGAUAUGGCAGGGAAGGG[2]

Morin (hEB) UGAGGAUAUGGCAGGGAAGGGGA[1]

Morin (hESC) UGAGGAUAUGGCAGGGAAGGGG[4]

Morin (hESC) UGAGGAUAUGGCAGGGAAG[1]

Morin (hESC) UGAGGAUAUGGCAGGGAAGG[1]

17 AAAAAAUCAUGAACUGACAACAGACUUAAUCUCAACUUCAAGUCCCGGCUUAGCCACCUAUUAAGUGACUUGAACUUGGGACUAAGCCUGUUGUCGGUUCAGCCCUCUAC

.........((((((((((((((.((((.((((((.((((((((...((((((.......)))))))))))))).)))))).)))).)))))))))))))).........

Morin (hESC) CUUGAACUUGGGACUAAGCCUG[2]

Morin (hESC) UUGAACUUGGGACUAAGCCUGUU[1]

Taft (FANTOM4) CUUGAACUUGGGACUAAGCCUG[5]

18 UAGUUUUCUUAAGUUUCCUCUGAAAACCCAAACACUUUUACAUAUGUAGAAGUUCUACUACACAUGUAGAAAUGUGUGGGUUUUCAGAGGAAACUUAAGAAAACUA

((((((((((((((((((((((((((((((.(((.((((((((.(((((........))))).)))))))).))).))))))))))))))))))))))))))))))

ENCODE (GSE14362) ACAUGUAGAAAUGUGUGGGUUUU[1]

ENCODE (GSE14362) AUGUGUGGGUUUUCAGAGGAAA[2]

Morin (hEB) ACAUGUAGAAAUGUGUGGGUU[1]

Morin (hEB) ACAUGUAGAAAUGUGUGGGUUUU[3]

Morin (hESC) ACAUGUAGAAAUGUGUGGGUUUU[3]

19 AUAACAUUGUCAGACGUGUCAUCCCCAGAUACAAUGGACAAUAUGCUAUUAUAAUCGUAUGGCAUUGUCCUUGCUGUUUGGAGAUAAUACUGCUGACUUUAU

........(((((..((((.(((.((((((((((.(((((..(((((((.........))))))))))))))).))))))).))).))))..))))).....

ENCODE (GSE14362) AUCCCCAGAUACAAUGGACAAU[1]

ENCODE (GSE14362) AUUGUCCUUGCUGUUUGGAGAU[3]

ENCODE (GSE14362) AUUGUCCUUGCUGUUUGGAGAUAA[2]

ENCODE (GSE14362) CAUUGUCCUUGCUGUUUGGA[1]

ENCODE (GSE14362) GUCCUUGCUGUUUGGAGAUAA[2]

ENCODE (GSE14362) UGUCCUUGCUGUUUGGAGA[1]

ENCODE (GSE14362) UGUCCUUGCUGUUUGGAGAU[2]

ENCODE (GSE14362) UGUCCUUGCUGUUUGGAGAUA[7]

ENCODE (GSE14362) UGUCCUUGCUGUUUGGAGAUAA[13]

ENCODE (GSE14362) UGUCCUUGCUGUUUGGAGAUAAU[1]

ENCODE (GSE14362) UUGUCCUUGCUGUUUGGAGAUA[3]

ENCODE (GSE14362) UUGUCCUUGCUGUUUGGAGAUAA[2]

Morin (hEB) AUCCCCAGAUACAAUGGACAAUAU[1]

Morin (hEB) AUUGUCCUUGCUGUUUGGAG[1]

Morin (hEB) AUUGUCCUUGCUGUUUGG[2]

Morin (hEB) AUUGUCCUUGCUGUUUGGAGAUAA[1]

Morin (hEB) UGUCCUUGCUGUUUGGAGAUAA[1]

Morin (hEB) AUCCCCAGAUACAAUGGAC[1]

Morin (hEB) AUCCCCAGAUACAAUGGACAAU[9]

Morin (hESC) UGUCCUUGCUGUUUGGAGAU[2]

Morin (hESC) UGUCCUUGCUGUUUGGAGAUA[1]

Morin (hESC) AUUGUCCUUGCUGUUUGG[3]

Morin (hESC) AUUGUCCUUGCUGUUUGGAG[3]

Morin (hESC) AUUGUCCUUGCUGUUUGGAGAU[1]

Morin (hESC) AUCCCCAGAUACAAUGGACAA[1]

Morin (hESC) AUCCCCAGAUACAAUGGACAAUA[1]

Morin (hESC) AUUGUCCUUGCUGUUUGGA[2]

Morin (hESC) AUCCCCAGAUACAAUGGACAAU[7]

Morin (hESC) AUUGUCCUUGCUGUUUGGAGAUA[1]

Taft (FANTOM4) AUCCCCAGAUACAAUGGACA[1]

Taft (FANTOM4) AUCCCCAGAUACAAUGGACAA[2]

Taft (FANTOM4) AUCCCCAGAUACAAUGGACAAU[31]

Taft (FANTOM4) AUUGUCCUUGCUGUUUGGAGA[2]

Taft (FANTOM4) AUUGUCCUUGCUGUUUGGAGAU[9]

Taft (FANTOM4) AUUGUCCUUGCUGUUUGGAGAUAA[4]

Taft (FANTOM4) GUCCUUGCUGUUUGGAGAU[1]

Taft (FANTOM4) UCCCCAGAUACAAUGGACAAU[1]

Taft (FANTOM4) UGUCCUUGCUGUUUGGAGA[7]

Taft (FANTOM4) UGUCCUUGCUGUUUGGAGAU[14]

Taft (FANTOM4) UGUCCUUGCUGUUUGGAGAUA[7]

Taft (FANTOM4) UGUCCUUGCUGUUUGGAGAUAA[22]

Taft (FANTOM4) UGUCCUUGCUGUUUGGAGAUAAU[2]

Taft (FANTOM4) UUGUCCUUGCUGUUUGGAGAUA[1]

Taft (FANTOM4) UUGUCCUUGCUGUUUGGAGAUAA[1]

Wyman (GSM379265) UGUCCUUGCUGUUUGGAGA[1]

Wyman (GSM379266) UGUCCUUGCUGUUUGGAGA[4]

20 AUCCAUGUCUAUGUAUAUCUACAUCUGUAUCUACCUAGGUAGAGCAAUAAUAGAUUUAUAAUAGAUCUACCAGGGUAGAUACAGAUGUAGAUAUACAUAUCUAUAUAU

....(((..(((((((((((((((((((((((((((.((((((...((((.....))))......)))))).)))))))))))))))))))))))))))..)))....

Morin (hEB) UACCAGGGUAGAUACAGAU[1]

Morin (hESC) UACCAGGGUAGAUACAGAUGU[2]

21 AGCUCAGGGCGGCUGCGCAGAGGGCUGGACUCAGCGGCGGAGCUGGCUGCUGGCCUCAGUUCUGCCUCUGUCCAGGUCCUUGUGACCCGCCCGCUCUCCU

......((((((...((((.((((((((((..((.((((((((((((.....)))..))))))))).))))))).)))))))))..))))))........

Zhu (GSE14738) UUCUGCCUCUGUCCAGGUCCU[2]

Morin (hEB) AGGGCUGGACUCAGCGGCGG[3]

Morin (hEB) UUCUGCCUCUGUCCAGGUCCUU[3]

Morin (hEB) AGGGCUGGACUCAGCGGCGGAGCUG[2]

Morin (hEB) AGGGCUGGACUCAGCGGCGGAGCUGGC[1]

Morin (hEB) GCUGCGCAGAGGGCUGGACU[1]

Morin (hEB) AGAGGGCUGGACUCAGCGG[1]

Morin (hEB) AGGGCUGGACUCAGCGGCGGAGCU[3]

Morin (hEB) AGGGCUGGACUCAGCGGCGGAG[1]

Morin (hESC) AGGGCUGGACUCAGCGGCGGAGCUG[1]

Morin (hESC) UUCUGCCUCUGUCCAGGUCCUU[2]

Morin (hESC) AGGGCUGGACUCAGCGGCGG[3]

Morin (hESC) CGGCUGCGCAGAGGGCUGGACUCAGC[1]

Morin (hESC) UGGACUCAGCGGCGGAGCU[1]

Morin (hESC) GGGCUGGACUCAGCGGCGGAG[1]

Morin (hESC) GCUGCGCAGAGGGCUGGACUC[1]

Morin (hESC) AGGGCUGGACUCAGCGGC[2]

Seila (GSE13483) AGGGCUGGACUCAGCGGCGGA[1]

Seila (GSE13483) AGGGCUGGACUCAGCGGCGGAGCU[6]

Seila (GSE13483) AGGGCUGGACUCAGCGGCGGAGCUG[3]

Seila (GSE13483) UGGACUCAGCGGCGGAGCU[1]

Taft (FANTOM4) UCUGCCUCUGUCCAGGUCCUU[1]

22 GACAGACGCUUGGACAGGCACCUGAGGCUCUGUUAGCCUUGGCUCUGGGUCCUGCUCCUUAGAGCAGAGGCAGAGAGGCUCAGGGUCUGUCUGGGUCACUCUCU

((.((..(((..(((((((.((((((.((((.(..(((((.((((((((........)))))))).)))))).)))).)))))))))))))..)))..)).)).

Morin (hEB) AGCAGAGGCAGAGAGGCUCAGG[2]

Morin (hESC) AGCAGAGGCAGAGAGGCUCAG[1]

Morin (hESC) AGCAGAGGCAGAGAGGCUC[1]

Taft (FANTOM4) UGAGGCUCUGUUAGCCUUGGCU[3]

Taft (FANTOM4) UGAGGCUCUGUUAGCCUUGGCUC[2]

Taft (FANTOM4) UGAGGCUCUGUUAGCCUUGGCUCU[1]

23 GGUGCUGACUAUCCAGUUGAUGGGCUGCCAGAUCUGGGGCAUGCCCAGGACCUCAUGGAUGGGCUGCCUCCAGGUGACAGCAAUCAGCUGGCCUGGUUUGAUACU

(((((.(((((.(((((((((..((((.((..(((((((((.(((((...((....)).))))))))).))))))).)))).)))))))))..))))).).))))

Morin (hEB) UGGGCUGCCAGAUCUGGGG[1]

Morin (hEB) UGGGCUGCCAGAUCUGGGGCAUGC[1]

Morin (hESC) UGGGCUGCCAGAUCUGGGGCAUG[1]

Morin (hESC) UGGGCUGCCAGAUCUGGGGCAUGC[1]

Morin (hESC) UGGGCUGCCAGAUCUGGGGCAU[1]

Morin (hESC) UGGGCUGCCAGAUCUGGGGCAUGCC[2]

24 CUCGCGGUCCAGACGUGGCGGGGGUGGCGGCGGCAUCCCGGACGGCCUGUGAGGGAUGCGCCGCCCACUGCCCCGCGCCGCCUGACCGUCCCC

...((((((.((..((((((((((((((((((.((((((..((.....))..)))))))))))))....))))).))))))))))))))....

Morin (hEB) GAUGCGCCGCCCACUGCC[1]

Morin (hEB) GAUGCGCCGCCCACUGCCCCGCGC[3]

Morin (hEB) GAUGCGCCGCCCACUGCCC[1]

Morin (hEB) GAUGCGCCGCCCACUGCCCCG[1]

Morin (hEB) GAUGCGCCGCCCACUGCCCCGCG[5]

Morin (hESC) GAUGCGCCGCCCACUGCC[1]

Morin (hESC) GAUGCGCCGCCCACUGCCCCGCGC[18]

Morin (hESC) GAUGCGCCGCCCACUGCCCC[1]

Morin (hESC) GAUGCGCCGCCCACUGCCC[3]

Morin (hESC) GAUGCGCCGCCCACUGCCCCG[1]

Morin (hESC) GAUGCGCCGCCCACUGCCCCGCGCC[1]

Morin (hESC) GAUGCGCCGCCCACUGCCCCGCG[16]

Morin (hESC) AUGCGCCGCCCACUGCCCCGC[1]

Morin (hESC) GAUGCGCCGCCCACUGCCCCGC[3]

Seila (GSE13483) GAUGCGCCGCCCACUGCCC[1]

Seila (GSE13483) GAUGCGCCGCCCACUGCCCCG[1]

Seila (GSE13483) GAUGCGCCGCCCACUGCCCCGC[5]

Seila (GSE13483) GAUGCGCCGCCCACUGCCCCGCG[7]

Seila (GSE13483) GAUGCGCCGCCCACUGCCCCGCGC[24]

Seila (GSE13483) GAUGCGCCGCCCACUGCCCCGCGCC[2]

Taft (FANTOM4) AUGCGCCGCCCACUGCCCC[1]

Taft (FANTOM4) AUGCGCCGCCCACUGCCCCGC[1]

Taft (FANTOM4) AUGCGCCGCCCACUGCCCCGCG[1]

Taft (FANTOM4) AUGCGCCGCCCACUGCCCCGCGC[3]

Taft (FANTOM4) AUGCGCCGCCCACUGCCCCGCGCC[1]

Taft (FANTOM4) GAUGCGCCGCCCACUGCCCCGC[1]

Taft (FANTOM4) GAUGCGCCGCCCACUGCCCCGCG[3]

Taft (FANTOM4) GAUGCGCCGCCCACUGCCCCGCGC[10]

Taft (FANTOM4) GCGGGGGUGGCGGCGGCAUCCC[4]

Wyman (GSM379267) GAUGCGCCGCCCACUGCCC[1]

25 GGAAUUUUUAACCCGAUCACUAGAUUAUCUACAAGGGAAUUUUUUUUUAAUUUAAAAAAUUCCCUUGUAGAUAACCCGGUGGUCAGGUUGGAUGGCUC

......(((((((.((((((..(.(((((((((((((((((((((........))))))))))))))))))))).)..)))))).)))))))......

Morin (hEB) AAUUCCCUUGUAGAUAACCCGGU[1]

Morin (hEB) AAUUCCCUUGUAGAUAACC[2]

Morin (hEB) AAUUCCCUUGUAGAUAACCCGG[5]

Morin (hESC) AGAUUAUCUACAAGGGAAUUUU[1]

Morin (hESC) AAUUCCCUUGUAGAUAACCC[2]

Morin (hESC) AUUCCCUUGUAGAUAACCCGG[1]

Morin (hESC) AAUUCCCUUGUAGAUAACCCGGU[7]

Morin (hESC) AAUUCCCUUGUAGAUAACCCGG[8]

Morin (hESC) AAUUCCCUUGUAGAUAACCCG[1]

26 AAGUAAAAAUUAAUAUGAAACUGACUGAAUAGGUAGGGUCAUUUUUCUGUGACUGCACAUGGCCCAACCUAUUCAGUUAGUUCCAUAUUAGUUUAUUU

......(((((((((((.((((((((((((((((.((((((......((((....)))))))))).)))))))))))))))).)))))))))))....

ENCODE (GSE14362) CUGACUGAAUAGGUAGGGUCA[2]

ENCODE (GSE14362) CUGACUGAAUAGGUAGGGUCAU[5]

ENCODE (GSE14362) UGACUGAAUAGGUAGGGUCAU[1]

ENCODE (GSE14362) UGACUGAAUAGGUAGGGUCAUUU[1]

Ender (GSM337571) CUGACUGAAUAGGUAGGGUC[1]

Morin (hEB) CUGACUGAAUAGGUAGGGUCAUU[1]

Morin (hEB) CUGACUGAAUAGGUAGGGUCAU[4]

Morin (hESC) CUGACUGAAUAGGUAGGGUC[1]

Morin (hESC) UGACUGAAUAGGUAGGGUC[1]

Taft (FANTOM4) CUGACUGAAUAGGUAGGGUCA[5]

Taft (FANTOM4) CUGACUGAAUAGGUAGGGUCAU[5]

Taft (FANTOM4) GGCCCAACCUAUUCAGUUAGUU[1]

Taft (FANTOM4) UGACUGAAUAGGUAGGGUCAUU[4]

Taft (FANTOM4) UGGCCCAACCUAUUCAGUUAGU[1]

27 UAUAAGUCUGGUAGAGUGAGCUCUAAUCCAAUAUUACUAGCUUCUUUAUAAGAAGAGGAAACUAGUAAUGUUGGAUUAGGGCUCACUCUACUAUUCUCAGC

....((..(((((((((((((((((((((((((((((((((((((((....)))))))...))))))))))))))))))))))))))))))))..))....

Morin (hEB) AACUAGUAAUGUUGGAUUAGGG[2]

Morin (hESC) ACUAGUAAUGUUGGAUUAGGG[1]

Morin (hESC) AACUAGUAAUGUUGGAUUAGGG[1]

28 UCUUCCUCUCUGUCCUCUGGAAUUUGGUUUCUGAGGCACUUAGUAGGUGAUAGCAUGACUGACUGCCUCACUGACCACUUCCAGAUGAGGGUUACUC

.........((.((.(((((((..(((((..(((((((.(((((..((....))...))))).)))))))..))))).))))))).)).))......

Morin (hESC) AAUUUGGUUUCUGAGGCACUUAGU[2]

Morin (hESC) AAUUUGGUUUCUGAGGCACUU[2]

29 UGUCCAACAGAAAGGGAUCUGGAGGUCAGAGUGGAUCUCACCUGCAUACUUACUCAGCUGGGAUCUCCACUCCUGCUUCUGCUCUUUCCGCACCGCA

.........(((((((...(((((((..((((((((((((.(((..........))).)))))..)))))))..)))))))))))))).........

Morin (hEB) UGGGAUCUCCACUCCUGCU[4]

Morin (hEB) UGGGAUCUCCACUCCUGCUU[24]

Morin (hEB) UGGGAUCUCCACUCCUGC[3]

Morin (hESC) UGGGAUCUCCACUCCUGCU[6]

Morin (hESC) UGGGAUCUCCACUCCUGCUU[37]

Morin (hESC) UGGGAUCUCCACUCCUGC[1]

30 UCAUAAUAAUGAAGUACAACUUCGGUAUACUUUGUGAAUUGGCUUUUACAAAAGACCAACUCACGAAGUAUACCGAAGUCAUACUUGAUUACAACUA

......((((.(((((..(((((((((((((((((((.((((((((....)))).)))).)))))))))))))))))))..))))).))))......

Morin (hEB) UUCGGUAUACUUUGUGAAUUGG[2]

Morin (hEB) UUCGGUAUACUUUGUGAAUU[1]

Morin (hESC) UUCGGUAUACUUUGUGAAUUGG[1]

31 CCUUCCCCUCCCCUCCUCGGCACUUCCCCCACCUCACUGCCCGGGUGCCCACAAGACUGUGGACAGUGAGGUAGAGGGAGUGCCGAGGAGGGCACAGCUGUG

..........(((((((((((((((((.(.((((((((((((((............))).)).))))))))).).)))))))))))))))))(((....)))

Morin (hEB) UGUGGACAGUGAGGUAGAGGGA[1]

Morin (hEB) ACAGUGAGGUAGAGGGAGUGCCG[1]

Morin (hEB) ACAGUGAGGUAGAGGGAGUG[2]

Morin (hEB) UGUGGACAGUGAGGUAGAGGG[1]

Morin (hESC) ACAGUGAGGUAGAGGGAGUG[1]

Morin (hESC) ACAGUGAGGUAGAGGGAGUGC[1]

Morin (hESC) UGUGGACAGUGAGGUAGAG[1]

Morin (hESC) AAGACUGUGGACAGUGAGGUA[2]

Taft (FANTOM4) ACAGUGAGGUAGAGGGAGUGC[1]

32 UUCUAUUGAGCUGACUGGCUUGUAUGGAGGUUCUAGACCAUGUUAGUGUUCAAGUCUACAUGGAUGGAAACCUUCAAGCAGGCCAAGCAGGAGACAGGUG

.(((.((...(((..((((((((.(((((((((((..((((((.((.........)))))))).))).)))))))).))))))))..)))..)).)))..

ENCODE (GSE14362) CAUGGAUGGAAACCUUCAAG[1]

ENCODE (GSE14362) UACAUGGAUGGAAACCUUCAAGC[1]

ENCODE (GSE14362) UAUGGAGGUUCUAGACCAUGUU[1]

Morin (hEB) AUGGAUGGAAACCUUCAAGC[1]

Morin (hEB) UACAUGGAUGGAAACCUUCAAGC[1]

Morin (hESC) UACAUGGAUGGAAACCUUCAAGCA[2]

Morin (hESC) ACAUGGAUGGAAACCUUCAAGCA[1]

Morin (hESC) UACAUGGAUGGAAACCUUCAAGC[1]

Taft (FANTOM4) UAUGGAGGUUCUAGACCAUGU[3]

Taft (FANTOM4) UAUGGAGGUUCUAGACCAUGUU[2]

33 AGCAUUUCUAUUAGGUUGGUGCAAAAGUGAUUGCAGUGUUUGCCAAUAAAAGUAAUGACAAAAACUGCAGUUACUUUUGCACCAGCCCAAUAUAUGCCAA

.((((...((((.((((((((((((((((((((((((.((((.((.((....)).)).)))).)))))))))))))))))))))))).)))).))))...

Morin (hEB) CAAAAACUGCAGUUACUUUUGC[2]

Morin (hESC) ACAAAAACUGCAGUUACUUUUG[2]

Morin (hESC) CAAAAACUGCAGUUACUUUUGC[5]

Morin (hESC) AAAAGUGAUUGCAGUGUU[1]

Morin (hESC) AAAAGUGAUUGCAGUGUUU[1]

Morin (hESC) AAAAGUGAUUGCAGUGUUUGCC[2]

Wyman (GSM379268) CAAAAACUGCAGUUACUUU[3]

34 UAGCAUUACCGAACAAUCUAAAGGACCUGUACUAGGUUUAACAUGUUGAGCAUUACUCAUGUUAGACCUAGUACACGUCCUUUAGAUUCUUUAAAAUUCCCA

..........(((.((((((((((((.((((((((((((((((((............)))))))))))))))))).)))))))))))).)))..........

ENCODE (GSE14362) UGUUAGACCUAGUACACGUCCU[1]

Morin (hEB) UUAGACCUAGUACACGUCCUU[1]

Morin (hEB) UGUUAGACCUAGUACACGUCCU[1]

Morin (hEB) UUAGACCUAGUACACGUCCUUU[1]

Morin (hESC) UUAGACCUAGUACACGUCCUU[2]

Morin (hESC) UUAGACCUAGUACACGUCCUUU[1]

Seila (GSE13483) UGUUAGACCUAGUACACGUCC[1]

Seila (GSE13483) UGUUAGACCUAGUACACGUCCU[2]

Seila (GSE13483) UUAGACCUAGUACACGUCCUU[1]

Taft (FANTOM4) UGUUAGACCUAGUACACGUCCU[1]

35 UUUGGUAUUUCACUUUUGGUAUUUUGUGCCUAAGCUCCUAAGUAUACUUACAGAAAAUAUUCUUAGGAGCUCAGGCACAAAAUACCAGAAGUUCUUUGAUGAA

...........((((((((((((((((((((.((((((((((.(((.((.....)).))).)))))))))).))))))))))))))))))))...........

Morin (hEB) UCUUAGGAGCUCAGGCACAAAA[1]

Morin (hEB) UUCUUAGGAGCUCAGGCACAA[1]

Morin (hEB) UAUUUCACUUUUGGUAUU[1]

Morin (hESC) UCUUAGGAGCUCAGGCACAAAAU[2]

Morin (hESC) UUCUUAGGAGCUCAGGCACAAA[1]

36 AAUACGAGAUCCAAAGACACUGCUUCCCUGAAGUCUACUCAAGUGAAGUAUGCAUCACUUGAGUAGACUUCAGGGAAGCAGUGUCUUUGGAUUCUAGUUA

...((.((((((((((((((((((((((((((((((((((((((((.(....).))))))))))))))))))))))))))))))))))))).))).))..

ENCODE (GSE14362) UUGAGUAGACUUCAGGGAAG[1]

Morin (hEB) UUGAGUAGACUUCAGGGAAGCA[1]

Morin (hESC) UUGAGUAGACUUCAGGGAAGCA[3]

Morin (hESC) UUGAGUAGACUUCAGGGAA[1]

37 UUUAUGUCCUCUUGAGGUACCUGAAUUACCAAAAGCUUUAUGUAUUCUGAAGUUAUUGAAAAUAAGAGCUUUUGGGAAUUCAGGUAGUUCAGGAGUGACUUUUCU

.....(((((((((((.((((((((((.(((((((((((.(((.(((..........))).)))))))))))))).)))))))))).)))))))).)))......

ENCODE (GSE14362) AGCUUUUGGGAAUUCAGGUAG[2]

Ender (GSM337571) AGCUUUUGGGAAUUCAGGU[1]

Morin (hEB) AGCUUUUGGGAAUUCAGGUAG[1]

Morin (hESC) AGCUUUUGGGAAUUCAGGUAGU[1]

Morin (hESC) GAGCUUUUGGGAAUUCAGGUA[1]

Morin (hESC) GAGCUUUUGGGAAUUCAGGUAG[1]

Morin (hESC) AGCUUUUGGGAAUUCAGGUA[1]

Morin (hESC) GCUUUUGGGAAUUCAGGUAG[1]

Morin (hESC) AGCUUUUGGGAAUUCAGGUAG[2]

Morin (hESC) GAGCUUUUGGGAAUUCAG[1]

38 AGAAAAAUGUUAGGGUGGUGCAAAAGUGAUCGUGGUUUUUGCAAUUUUUUAAUGACAAAAACCACAAUUACUUUUGCACCAACCUAACCUUGUU

........((((((.(((((((((((((((.((((((((((..(((....)))..)))))))))).))))))))))))))).))))))......

ENCODE (GSE14362) AAAAACCACAAUUACUUUU[1]

ENCODE (GSE14362) AAAAACCACAAUUACUUUUGCA[2]

ENCODE (GSE14362) AAAACCACAAUUACUUUUG[1]

ENCODE (GSE14362) AAACCACAAUUACUUUUGCA[1]

Morin (hEB) AAAAGUGAUCGUGGUUUUUGCA[1]

Morin (hEB) AACCACAAUUACUUUUGCACCA[1]

Morin (hEB) CAAAAGUGAUCGUGGUUUUUGC[1]

Morin (hEB) UGCAAAAGUGAUCGUGGUUUUUG[1]

Morin (hEB) AAACCACAAUUACUUUUGCACCA[1]

Morin (hEB) AAAAACCACAAUUACUUUUGC[1]

Morin (hEB) UGCAAAAGUGAUCGUGGUUUUUGC[1]

Morin (hEB) AAAAACCACAAUUACUUUUGCA[1]

Morin (hEB) CAAAAGUGAUCGUGGUUUUUG[4]

Morin (hESC) AAAAGUGAUCGUGGUUUUUGCA[1]

Morin (hESC) AAAACCACAAUUACUUUUGCAC[1]

Morin (hESC) ACCACAAUUACUUUUGCACCA[1]

Morin (hESC) CAAAAGUGAUCGUGGUUUUUG[11]

Morin (hESC) CAAAAGUGAUCGUGGUUUUUGCA[1]

Taft (FANTOM4) AAAAACCACAAUUACUUUUGC[2]

Taft (FANTOM4) AAAAACCACAAUUACUUUUGCAC[1]

Taft (FANTOM4) AAAACCACAAUUACUUUUGC[1]

Taft (FANTOM4) AAAACCACAAUUACUUUUGCA[1]

Taft (FANTOM4) AAACCACAAUUACUUUUGCAC[1]

Taft (FANTOM4) AAACCACAAUUACUUUUGCACC[1]

Taft (FANTOM4) AACCACAAUUACUUUUGCACC[1]

Taft (FANTOM4) CAAAAACCACAAUUACUUUUGC[2]

Taft (FANTOM4) CAAAAGUGAUCGUGGUUUUUGC[1]

Wyman (GSM379265) ACAAAAACCACAAUUACUU[3]

Wyman (GSM379265) CAAAAACCACAAUUACUUU[2]

Wyman (GSM379266) AACCACAAUUACUUUUGCA[1]

Wyman (GSM379267) CAAAAACCACAAUUACUUU[2]

Wyman (GSM379267) ACAAAAACCACAAUUACUU[1]

Wyman (GSM379268) AAAACCACAAUUACUUUUG[1]

Wyman (GSM379268) CAAAAACCACAAUUACUUU[2]

39 CCAUACUGGGGACUGCUCUUUAAAAGGCUGUUAAAUGGUGAUACUAUAUUUUUUAACAUCAACAUCAUUUAGAAACCUUUUAGAGAACAGUUCUCUUCACC

.......((((((((.(((((((((((...((((((((((......................))))))))))...))))))))))).))))))))......

ENCODE (GSE14362) UAAAAGGCUGUUAAAUGGUGAU[1]

Morin (hEB) AAAAGGCUGUUAAAUGGUGAUAC[1]

Morin (hEB) UAAAAGGCUGUUAAAUGGUGAU[4]

Morin (hEB) UAAAAGGCUGUUAAAUGGU[1]

Morin (hESC) UAAAAGGCUGUUAAAUGGUGAU[1]

Morin (hESC) UAAAAGGCUGUUAAAUGGU[1]

Morin (hESC) UAAAAGGCUGUUAAAUGGUG[1]

Morin (hESC) AAAAGGCUGUUAAAUGGUGAU[1]

40 GAAAGAAGAACUGGACAAAAUUAAAAUGCUCUUCUGUCAUUGUAAUAGUUCAUAUGGGCACUGACAGGAGAGCAUUUUGACUUUGUCAAGUGUGUCUGCU

...(((...(((.((((((.(((((((((((((((((((.(((.(((.....)))..))).))))))))))))))))))).)))))).)))...)))...

Morin (hEB) ACUGACAGGAGAGCAUUUUG[1]

Morin (hEB) CACUGACAGGAGAGCAUUUUGA[2]

Morin (hEB) ACUGACAGGAGAGCAUUUU[2]

Morin (hEB) ACUGACAGGAGAGCAUUUUGA[6]

Morin (hEB) CACUGACAGGAGAGCAUUUU[1]

Morin (hESC) ACUGACAGGAGAGCAUUUUGA[9]

Morin (hESC) ACUGACAGGAGAGCAUUUU[1]

Morin (hESC) CACUGACAGGAGAGCAUUUUGA[6]

Morin (hESC) ACUGACAGGAGAGCAUUUUG[4]

Morin (hESC) ACUGACAGGAGAGCAUUUUGAC[1]

Morin (hESC) CUGACAGGAGAGCAUUUUGA[1]

41 CACCUUCUCGCAGAGGCUCUUGACCUGGGACUCGGACAGCUGCUUGCACUCGUUCAGCUGCUCGAUCCACUGGUCCAGCUCCUUGGUGAACACCUU

.......((((.((((..((.((((..(((.(((..((((((..((....))..))))))..))))))...)))).))..)))).)))).......

Morin (hEB) UUGACCUGGGACUCGGACAGCU[1]

Morin (hEB) UGACCUGGGACUCGGACAG[1]

Morin (hEB) UGACCUGGGACUCGGACAGCUG[6]

Morin (hEB) UGACCUGGGACUCGGACAGCU[1]

Morin (hESC) UGACCUGGGACUCGGACAGCUGC[1]

Morin (hESC) UGACCUGGGACUCGGACAGCUG[10]

Morin (hESC) UGACCUGGGACUCGGACAGCU[1]

Morin (hESC) GACCUGGGACUCGGACAGCUG[1]

Seila (GSE13483) UGACCUGGGACUCGGACAG[1]

Seila (GSE13483) UGACCUGGGACUCGGACAGCU[8]

Seila (GSE13483) UGACCUGGGACUCGGACAGCUG[15]

42 UGAACAGUUAAAUUAUAACAUGUCCAUAUUAUGGGUUAGUUGUGGACACAUACUAACGCAUAAUAUGGACAUGUUAUAAUUUAACUGUUCCUUU

.((((((((((((((((((((((((((((((((.((((((.(((....))))))))).))))))))))))))))))))))))))))))))....

ENCODE (GSE14362) AACGCAUAAUAUGGACAUGU[1]

ENCODE (GSE14362) UAACGCAUAAUAUGGACAUGUUA[1]

ENCODE (GSE14362) UGUCCAUAUUAUGGGUUAGU[1]

Morin (hEB) UAACGCAUAAUAUGGACAUGUU[2]

Morin (hEB) AACGCAUAAUAUGGACAUGUUAU[1]

Morin (hEB) UAACGCAUAAUAUGGACAUGUUAU[2]

Morin (hEB) UAACGCAUAAUAUGGACAUGU[2]

Morin (hESC) AACGCAUAAUAUGGACAUGUUA[1]

Morin (hESC) UAACGCAUAAUAUGGACAUGUUAU[1]

Seila (GSE13483) AUAAUAUGGACAUGUUAUAAU[2]

Seila (GSE13483) AUACUAACGCAUAAUAUGG[1]

Seila (GSE13483) AUACUAACGCAUAAUAUGGACA[2]

Seila (GSE13483) UAACGCAUAAUAUGGACAUGU[2]

Seila (GSE13483) UAACGCAUAAUAUGGACAUGUU[1]

Taft (FANTOM4) ACGCAUAAUAUGGACAUGUUAU[2]

Taft (FANTOM4) UAACGCAUAAUAUGGACAUGU[2]

Taft (FANTOM4) UAACGCAUAAUAUGGACAUGUUA[1]

43 AUGAGUUUGCUUUGUGUCAUCCUCACAACAACCUUGCAGGGUAGAGAUGAUUUUUCCUACUUUUCUAGGUUGUUGGGGGCUGGGGCAGGGGGAACAGAG

....((((.((((((.(((.((((.(((((((((.(.(((((((.((.......))))))))).).))))))))))))).))).)))))).))))....

ENCODE (GSE14362) UCACAACAACCUUGCAGGGUA[1]

Morin (hESC) UACUUUUCUAGGUUGUUGGGGG[2]

44 GGCACUUGCUUGGGGGUUAGUGAGGACAGGGCAAAUUCACGAGAUUGGGUUGUGCAGAGGCUGACACUUGGAUUUUCCUGGGCCUCAGGACUUCCUUUCAGACAUGG

(((....))).(((((((..(((((.(((((.(((((((.(.(.(..(.((......)).)..)).).))))))))))))..))))).)))))))............

Morin (hEB) CUUGGAUUUUCCUGGGCCUCAG[1]

Morin (hEB) UGAGGACAGGGCAAAUUCACGA[1]

Morin (hESC) CUUGGAUUUUCCUGGGCCUCAG[1]

Morin (hESC) UGAGGACAGGGCAAAUUCACGA[4]

45 CAGUUUCAACCUGAGAUCUAAUCCUUUCAGCUCUACAACUCAGUGUCUUGUUUUUAGGUAGUGAGUUGUAGAGCUGGAAGGGUUGGAUCUGGUAAAAAGAAUCGAAAU

........(((..(((((((((((((((((((((((((((((.(..((.......))..).))))))))))))))))))))))))))))))))...............

ENCODE (GSE14362) UAGUGAGUUGUAGAGCUGGAA[1]

Morin (hEB) UGAGUUGUAGAGCUGGAAGGGU[2]

Morin (hESC) AGUUGUAGAGCUGGAAGGGUU[1]

Morin (hESC) UGAGUUGUAGAGCUGGAAGGGUU[1]

46 GUGAAUAUUGAGGCACUGGGUAGUGGAUGAUGGAGACUCGGUACCCACUGCUGAGGGUGGGGACCAAGUCUGCGUCAUCCUCUCCUCAGUGCCUCAAACGGUC

.......(((((((((((((.((.((((((((.(((((.(((.((((((......)))))).))).))))).)))))))).)).)))))))))))))......

ENCODE (GSE14362) AUGAUGGAGACUCGGUACC[1]

ENCODE (GSE14362) CCAAGUCUGCGUCAUCCUCU[1]

ENCODE (GSE14362) UAGUGGAUGAUGGAGACUCGG[1]

ENCODE (GSE14362) UAGUGGAUGAUGGAGACUCGGU[1]

ENCODE (GSE14362) UGGGGACCAAGUCUGCGUCAU[1]

Ender (GSM337571) AGUGGAUGAUGGAGACUCGGU[1]

Morin (hEB) UAGUGGAUGAUGGAGACUCGG[1]

Morin (hEB) GUGGAUGAUGGAGACUCGGUACCC[1]

Morin (hEB) ACCAAGUCUGCGUCAUCCUCUC[1]

Morin (hEB) UGGAUGAUGGAGACUCGGUAC[1]

Morin (hEB) AGUGGAUGAUGGAGACUCGG[1]

Morin (hEB) AGUGGAUGAUGGAGACUCGGUA[1]

Morin (hESC) UAGUGGAUGAUGGAGACUC[3]

Morin (hESC) UAGUGGAUGAUGGAGACUCGG[3]

Morin (hESC) CCAAGUCUGCGUCAUCCUCUC[1]

Morin (hESC) AGUGGAUGAUGGAGACUCGGUAC[1]

Morin (hESC) AGUGGAUGAUGGAGACUCGGUACC[1]

Morin (hESC) UAGUGGAUGAUGGAGACUCGGU[3]

Morin (hESC) GUGGAUGAUGGAGACUCGGU[1]

Morin (hESC) GUGGAUGAUGGAGACUCGG[1]

Morin (hESC) UGGAUGAUGGAGACUCGGUACC[1]

Morin (hESC) AUGAUGGAGACUCGGUACCCA[1]

Morin (hESC) UGAUGGAGACUCGGUACCCACU[1]

Morin (hESC) UAGUGGAUGAUGGAGACUCGGUA[2]

47 UCAGACUGAAACUACACUUUAAGGGGACCAAAGAGAUAUAUAGAUAUCAGCUACCUAUAUACCUGUUCGGUCUCUUUAAAGUGUAGUUUAACUGAUU

((((....(((((((((((((((((((((.((.((.(((((((...........))))))).)).)).)))))))))))))))))))))..))))..

ENCODE (GSE14362) AAGGGGACCAAAGAGAUAUAU[1]

ENCODE (GSE14362) AAGGGGACCAAAGAGAUAUAUAGA[1]

ENCODE (GSE14362) AUACCUGUUCGGUCUCUUUA[1]

ENCODE (GSE14362) AUAUACCUGUUCGGUCUCUUU[9]

ENCODE (GSE14362) AUAUACCUGUUCGGUCUCUUUA[5]

ENCODE (GSE14362) UAUACCUGUUCGGUCUCUUU[3]

ENCODE (GSE14362) UAUACCUGUUCGGUCUCUUUA[2]

ENCODE (GSE14362) UAUACCUGUUCGGUCUCUUUAA[2]

ENCODE (GSE14362) UAUAUACCUGUUCGGUCUCUUU[2]

Morin (hESC) UAUACCUGUUCGGUCUCUUUAA[1]

Morin (hESC) AAGGGGACCAAAGAGAUAUAUA[2]

Morin (hESC) AUAUACCUGUUCGGUCUCUUUA[1]

Morin (hESC) AGGGGACCAAAGAGAUAUAUAG[3]

48 UGUGUUUUCCUCAACGCUCACAGUUACACUUCUUACUCUCAAUCCAUUCAUAUUGAAAAUGAUGAGUAGUGACUGAUGAAGCACAAAUCAGCCAA

((((((...........((((((((((((((.(((.(.(((((........))))).).))).)))).))))))).)))))))))..........

ENCODE (GSE14362) GAAAAUGAUGAGUAGUGACUGAUG[1]

ENCODE (GSE14362) UGAAAAUGAUGAGUAGUGACUGAUG[1]

Morin (hEB) GAAAAUGAUGAGUAGUGACUGAUG[8]

Morin (hEB) UGAAAAUGAUGAGUAGUGACUG[1]

Morin (hEB) UGAAAAUGAUGAGUAGUGACUGAUG[2]

Morin (hEB) UGAAAAUGAUGAGUAGUG[1]

Morin (hESC) GAAAAUGAUGAGUAGUGACUGAUGA[1]

Morin (hESC) GAAAAUGAUGAGUAGUGACU[1]

Morin (hESC) GAAAAUGAUGAGUAGUGACUGAUG[9]

Morin (hESC) GAAAAUGAUGAGUAGUGACUGAU[4]

Morin (hESC) UGAAAAUGAUGAGUAGUGACUG[3]

Morin (hESC) UGAAAAUGAUGAGUAGUGACUGAUG[3]

Morin (hESC) UGAAAAUGAUGAGUAGUGACUGAU[3]

Morin (hESC) GAAAAUGAUGAGUAGUGACUG[11]

Morin (hESC) UGAAAAUGAUGAGUAGUGACU[1]

Morin (hESC) AAUGAUGAGUAGUGACUGA[1]

49 AUUUGUUUAUAUGAGUUCAACUCCAAACACUCAAAACUCAUUGUUGAAUGGAAUGAGAUAUUUUGAGUGUUUGGAAUUGAACUCGUAUACACUGAU

....((.(((((((((((((.(((((((((((((((((((((.(.....).))))))...))))))))))))))).))))))))))))).))....

ENCODE (GSE14362) AACUCCAAACACUCAAAACUCA[1]

ENCODE (GSE14362) AGAUAUUUUGAGUGUUUGGAAU[1]

ENCODE (GSE14362) AGAUAUUUUGAGUGUUUGGAAUU[1]

ENCODE (GSE14362) AGAUAUUUUGAGUGUUUGGAAUUG[2]

ENCODE (GSE14362) GAUAUUUUGAGUGUUUGGAAUU[2]

ENCODE (GSE14362) GAUAUUUUGAGUGUUUGGAAUUG[1]

ENCODE (GSE14362) UAUUUUGAGUGUUUGGAAUUG[1]

ENCODE (GSE14362) UAUUUUGAGUGUUUGGAAUUGA[2]

Morin (hEB) AACUCCAAACACUCAAAACUCA[1]

Morin (hEB) UAUUUUGAGUGUUUGGAAUUG[1]

Morin (hEB) AGAUAUUUUGAGUGUUUGGAAUUG[2]

Morin (hEB) AGAUAUUUUGAGUGUUUGGA[1]

Morin (hESC) AGAUAUUUUGAGUGUUUGGAAUUG[4]

50 UUGCUUUUAUUAGGUUGCUGCAAAACUAAUUGCAGUUUUCCAGUUUUGUUUUAAUUGCAAAACUGUAAUUACUUUUUUACCAAUCCAAUAAAUAGAG

...((((((((.(((((.((.((((.(((((((((((((.(((((.......))))).))))))))))))).)))).)).))))).)))))).))..

Morin (hEB) AAACUGUAAUUACUUUUU[8]

Morin (hEB) AAAACUGUAAUUACUUUUU[13]

Morin (hESC) AAACUGUAAUUACUUUUU[23]

Morin (hESC) AAAACUGUAAUUACUUUUU[11]

51 UACACAUUUUCUUUGCUAAGUCCCUUCUUUCUAUCCUAGUAUAACUUGAAGAAUUCAAAUAGUCAUGCUAGGAUAGAAAGAAUGGGACUUGGCCAGGGAAGAAGAGU

......((((((((((((((((((((((((((((((((((((.((((((.....)))...))).)))))))))))))))))).))))))))).))))))))).....

Morin (hEB) CAUGCUAGGAUAGAAAGAAUG[1]

Morin (hEB) CAUGCUAGGAUAGAAAGAAUGG[1]

Morin (hEB) CAUGCUAGGAUAGAAAGAAUGGG[1]

Morin (hESC) UCAUGCUAGGAUAGAAAGAAUGG[1]

Morin (hESC) AUGCUAGGAUAGAAAGAAUGG[1]

Morin (hESC) CAUGCUAGGAUAGAAAGAAUGGGA[2]

52 CACAGACGGCAGCUGCGGCCUAGCCCCCAGGCUUCACUUGGCGUGGACAACUUGCUAAGUAAAGUGGGGGGUGGGCCACGGCUGGCUCCUACCU

....((.(.((((((.((((((.(((((..((((.((((((((.(.....).)))))))).))))))))).)))))).)))))).)))......

Morin (hEB) UAGCCCCCAGGCUUCACUUGGCGU[1]

Morin (hESC) UAAGUAAAGUGGGGGGUGGG[4]

Morin (hESC) UAAGUAAAGUGGGGGGUGG[1]

Morin (hESC) UAGCCCCCAGGCUUCACUUGGCG[5]

Morin (hESC) CUAAGUAAAGUGGGGGGUGGGC[1]

53 UCGUUUGACUCUAUUGGACGAGGGGACUGGUUAAUAGAACUAACUAACCAGAACUAUUUUGUUCUGUUAACCCAUCCCCUCAUCUAAUAGAGUAUAAACC

..((((((((((((((((.(((((((..((((((((((((....................))))))))))))..))))))).))))))))))).))))).

Morin (hEB) AGGGGACUGGUUAAUAGAACUA[2]

Morin (hESC) AGGGGACUGGUUAAUAGAACUA[2]

54 UGUUGCCGUCCACCGUCGGGGCGCCUCCCACGCACGCUGCUGCGGGGGGCCUGGGCCACGCAGGGGUCGGGUGUGGGGGGUCCCGGGCAGGUGGUAGCGGCGUCU

....(((((((((((((.(((.(((((((((((.((((.(((((...(((....))).))))).)).)).)))))))))))))).))).)))))..)))))....

Morin (hESC) UCGGGUGUGGGGGGUCCCGGG[6]

55 UUUGCCAUGUUGGCCAGGCUGGUGUCAAACUCCUAGCUCAGGUGAUCCGCCUAGUGGUUAGGAUUUGGCGCUAUCUCUCCCUUUUGGUUUUG

...((((....((..(((.((((((((((.((((((((((((((...))))).).)))))))))))))))))))))..))....))))....

ENCODE (GSE14362) UAGUGGUUAGGAUUUGGCG[1]

Morin (hEB) GUGGUUAGGAUUUGGCGCU[34]

Morin (hEB) UAGUGGUUAGGAUUUGGCGCU[31]

Morin (hEB) UAGUGGUUAGGAUUUGGC[39]

Morin (hEB) AGUGGUUAGGAUUUGGCGC[5]

Morin (hEB) UUAGGAUUUGGCGCUAUC[1]

Morin (hEB) UAGUGGUUAGGAUUUGGCG[49]

Morin (hEB) AGUGGUUAGGAUUUGGCG[25]

Morin (hEB) UAGUGGUUAGGAUUUGGCGC[3]

Morin (hEB) UGGUUAGGAUUUGGCGCU[28]

Morin (hEB) AGUGGUUAGGAUUUGGCGCU[21]

Morin (hEB) GUGGUUAGGAUUUGGCGC[12]

Morin (hESC) UAGUGGUUAGGAUUUGGCG[154]

Morin (hESC) AGUGGUUAGGAUUUGGCGC[6]

Morin (hESC) UAGUGGUUAGGAUUUGGC[81]

Morin (hESC) UAGUGGUUAGGAUUUGGCGCU[149]

Morin (hESC) CCUAGUGGUUAGGAUUUGGCGCU[1]

Morin (hESC) CUAGUGGUUAGGAUUUGGCGCU[6]

Morin (hESC) GUGGUUAGGAUUUGGCGCU[74]

Morin (hESC) AGUGGUUAGGAUUUGGCG[65]

Morin (hESC) UAGUGGUUAGGAUUUGGCGC[23]

Morin (hESC) UGGUUAGGAUUUGGCGCU[64]

Morin (hESC) GUUGGCCAGGCUGGUGUCAAACUCC[1]

Morin (hESC) AGUGGUUAGGAUUUGGCGCU[78]

Morin (hESC) UGGUUAGGAUUUGGCGCUA[1]

Morin (hESC) CUAGUGGUUAGGAUUUGGCG[3]

Morin (hESC) CUAGUGGUUAGGAUUUGG[2]

Morin (hESC) GUGGUUAGGAUUUGGCGC[23]

56 CCCAAAAUUUAAGAAGGCCCUCACUCUCAGGGUUGUACAAGAGCAGGGGUGGCACUUGUACAGCCCUGAGAGUGAGGACCUCCUUAAAUUUUGUA

..(((((((((((.(((.(((((((((((((((((((((((.((.......)).))))))))))))))))))))))).))).)))))))))))..

Morin (hESC) ACUUGUACAGCCCUGAGAGUGA[2]

Taft (FANTOM4) CUUGUACAGCCCUGAGAGUGAGG[1]

57 GAUACGGGGGCCAUGAGGGUGGGGUCCAGGCUGGACCAGGCCUGCCCUGAGUCCCCCAGCAGGUGCUCCAGGCUGGCUCACACCCUCUGCCUCUCUCUCUU

((.(.((((((...((((((((((.((((.(((((.(..((((((..((.......))))))))).))))).))))))).))))))).)))))).).))..

Morin (hEB) GGUGCUCCAGGCUGGCUCACA[1]

Morin (hESC) AGGUGCUCCAGGCUGGCUCACA[1]

Morin (hESC) AGGUGCUCCAGGCUGGCUCAC[1]

Morin (hESC) GGUGCUCCAGGCUGGCUCACA[1]

Morin (hESC) GGUGCUCCAGGCUGGCUCAC[2]

58 AACUAUUCUUAGGUUGAUGCAGAAGUAACUACGGUUUUUGCAGUUGAAAGUAAUGGCAAAGACCGUGACUACUUUUGCAACAGCCUAAUAGUUUCU

((((((...(((((((.((((((((((..((((((((((((.((((....)))).))))))))))))..)))))))))).)))))))))))))...

Morin (hEB) AAAGACCGUGACUACUUUU[4]

Morin (hEB) AAAGACCGUGACUACUUUUGCA[1]

Morin (hESC) AAAGACCGUGACUACUUUUGC[2]

Morin (hESC) AAAGACCGUGACUACUUUUGCA[1]

Morin (hESC) AGAAGUAACUACGGUUUUUGCA[1]

59 GACGGUGGAGGGGCCGACGCGGAGAGCGGCUCUAGGUGGGUUUGGCGGCGGCGAGGACACCGCCGCUCCCUCUAGGGUCGCUCGGAGCGUGACCCUGAGAACUCUC

((.(((...((((.(.((((...(((((((((((((.(((...((((((((.(....).))))))))))))))))))))))))...))))).))))....))).))

Morin (hEB) GCUCCCUCUAGGGUCGCUCGGA[1]

Morin (hEB) GCUCCCUCUAGGGUCGCUCGG[1]

Morin (hESC) GCUCCCUCUAGGGUCGCUCGGA[2]

Morin (hESC) GCUCCCUCUAGGGUCGCUCGG[2]

Seila (GSE13483) GCUCCCUCUAGGGUCGCUCGGA[6]

60 GGAGUGGGGUAGAGGCAGGGAAUCCAGGACUAACUACACUUAAUUUUCUUGAAUUAAUUAAGUGUAGUUAGUCCUGGAUUCCCUGCCUCUUAUUCUUGAA

.....((((((((((((((((((((((((((((((((((((((((..........)))))))))))))))))))))))))))))))))).))))))....

Morin (hEB) UCCAGGACUAACUACACUUAAU[2]

Morin (hEB) UAAGUGUAGUUAGUCCUGGAUU[3]

Morin (hEB) UCCAGGACUAACUACACUUA[2]

61 AAAUGGGAUAUACAUACAUGUACACACACAUGUCAUCCACACACAUACAUAUAUAUAUGUUUGUAUGGAUAUGUGUGUGUAUGUGUGUGUAUACACAGAA

.....(..((((((((((((((((((((((((((....(((.(((((........))))).)))...))))))))))))))))))))))))))..)....

Morin (hEB) UUUGUAUGGAUAUGUGUGUGUAU[2]

Morin (hEB) UUUGUAUGGAUAUGUGUGU[3]

Morin (hESC) UUGUAUGGAUAUGUGUGUGUAU[1]

Morin (hESC) UUUGUAUGGAUAUGUGUGU[2]

Taft (FANTOM4) GUAUGGAUAUGUGUGUGUAU[1]

62 AACAAGCCUCACUAGGCUAAUGGGGGGCAGGGAGCGAGGCCGGGAAAUAGGGGCAUAUGCCUCGUCUUUGUUCUCUUUCCUCCCAGCAUGCCAGUGCAGCAGGGC

.....((((((((.(((...(((((((.(((((((((((.((((..(((......)))..)))).)))))))))))..)))))))....))))))).....))))

Morin (hEB) UGGGGGGCAGGGAGCGAGGC[3]

Morin (hEB) UGGGGGGCAGGGAGCGAG[8]

Morin (hESC) UGGGGGGCAGGGAGCGAGGC[4]

Morin (hESC) UGGGGGGCAGGGAGCGAGG[1]

Morin (hESC) UGGGGGGCAGGGAGCGAG[17]

63 UGCCUAGCUCUUCUUCUCCCCUGUCCUUGCUUUGGAGCAGGCUAGGUGGCUCCCACUUCUUGGCCUGUUAGAGCAGGGACUGGGGUGGGGAGGCCGAGGGG

..(((....(((((((.((((.((((((((((((..(((((((((((((...)))))...)))))))))))))))))))).)))).)))))))...)))..

Morin (hEB) GGCCUGUUAGAGCAGGGACUG[1]

Morin (hESC) UGGCCUGUUAGAGCAGGGACUG[5]

64 GCUUCUGUACCCCUGCCCCAACAAGGAAGGACAAGAGGUGUGAGCCACACACACGCCUGGCCUCCUGUCUUUCCUUGUUGGAGCAGGGAUGUAGAAGC

(((((((((.((((((.((((((((((((((((.(((((..(.((.........)))..))))).)))))))))))))))).)))))).)))))))))

ENCODE (GSE14362) ACAAGGAAGGACAAGAGGUGUG[1]

ENCODE (GSE14362) UGCCCCAACAAGGAAGGACAAGA[1]

Ender (GSM337571) UGCCCCAACAAGGAAGGAC[1]

Morin (hEB) UGUCUUUCCUUGUUGGAGCAGG[1]

Morin (hEB) AAGGAAGGACAAGAGGUGUGAG[1]

Morin (hEB) ACAAGGAAGGACAAGAGGUGUGA[2]

Morin (hEB) ACAAGGAAGGACAAGAGGUGUGAG[1]

Morin (hEB) ACAAGGAAGGACAAGAGGUGUGAGC[1]

Morin (hEB) UGCCCCAACAAGGAAGGACAAG[1]

Morin (hESC) CAAGGAAGGACAAGAGGUGUGAG[1]

Morin (hESC) ACAAGGAAGGACAAGAGGUGUGA[3]

Morin (hESC) ACAAGGAAGGACAAGAGGUGUGAG[2]

Morin (hESC) ACAAGGAAGGACAAGAGGUGUG[1]

Morin (hESC) UGCCCCAACAAGGAAGGACAAGA[1]

65 GAGAUGCCUGUUCCGGGCAUCACCUCCCACUGCAGAGCCUGGGGAGCCGGACAGCUCCCUUCCCAGGCUCUGCAGUGGGAACUGAUGCCUGGAACAGUUCCUGC

.......(((((((((((((((..((((((((((((((((((((((..(((....)))))))))))))))))))))))))..))))))))))))))).......

Morin (hEB) CCAGGCUCUGCAGUGGGAA[1]

Morin (hEB) CCUCCCACUGCAGAGCCUGG[2]

Morin (hESC) CCAGGCUCUGCAGUGGGAACUG[2]

Morin (hESC) CCUCCCACUGCAGAGCCUGGGG[1]

Morin (hESC) CUCCCACUGCAGAGCCUGGGGA[1]

Morin (hESC) CCUCCCACUGCAGAGCCUGG[2]

Taft (FANTOM4) CCAGGCUCUGCAGUGGGAAC[2]

Taft (FANTOM4) CCUCCCACUGCAGAGCCUGGGGA[4]

Taft (FANTOM4) UCCCACUGCAGAGCCUGGGGAG[1]

Taft (FANTOM4) UCCCAGGCUCUGCAGUGGGAAC[2]

66 UUCUACAUCCUAUCCUCAGAUAGUGUUUAGGGUUCUGCUUGACCUCUUAAAAAGAACUUAUAUAUGCAAGCAAGGGCUGAUAUUCUGGGGGAGGUGCAUGCAU

.....((.(((.((((((((..(((((...(((((((((((......(((.......)))......))))).)))))))))))))))))))))))).......

Morin (hEB) UAGUGUUUAGGGUUCUGC[2]

Morin (hEB) UAGUGUUUAGGGUUCUGCUU[1]

Morin (hEB) UAGUGUUUAGGGUUCUGCU[1]

Morin (hESC) UAGUGUUUAGGGUUCUGC[7]

Morin (hESC) UAGUGUUUAGGGUUCUGCUU[3]

Morin (hESC) UAGUGUUUAGGGUUCUGCU[4]

67 UGAUUACAUUAUUCAGGCCGGUCCUGCAGAGAGGAAGCCCUUCCAAUACCUGUAAGCAGAAGGGCUUCCUCUCUGCAGGACCGGCCUGAAUAAUGAUCCU

......(((((((((((((((((((((((((((((((((((((...............))))))))))))))))))))))))))))))))))))).....

ENCODE (GSE14362) AGGGCUUCCUCUCUGCAGGAC[1]

Morin (hEB) AAGGGCUUCCUCUCUGCAGGA[4]

Morin (hEB) AAGGGCUUCCUCUCUGCA[4]

Morin (hEB) AAGGGCUUCCUCUCUGCAG[1]

Morin (hEB) AAGGGCUUCCUCUCUGCAGG[2]

Morin (hEB) AGGGCUUCCUCUCUGCAGG[1]

Morin (hEB) AAGGGCUUCCUCUCUGCAGGAC[17]

Morin (hEB) GAAGGGCUUCCUCUCUGCAGG[1]

Morin (hEB) GUCCUGCAGAGAGGAAGCCCUU[1]

Morin (hESC) AGGGCUUCCUCUCUGCAGGACCG[1]

Morin (hESC) AAGGGCUUCCUCUCUGCAGGA[6]

Morin (hESC) AAGGGCUUCCUCUCUGCAGG[5]

Morin (hESC) AAGGGCUUCCUCUCUGCAGGAC[29]

Morin (hESC) AAGGGCUUCCUCUCUGCAGGACC[1]

Taft (FANTOM4) AAGGGCUUCCUCUCUGCAGGAC[8]

Taft (FANTOM4) AAGGGCUUCCUCUCUGCAGGACC[1]

Taft (FANTOM4) AGGGCUUCCUCUCUGCAGGAC[4]

Taft (FANTOM4) GGGCUUCCUCUCUGCAGGACC[2]

Taft (FANTOM4) GGGCUUCCUCUCUGCAGGACCG[1]

Taft (FANTOM4) GGUCCUGCAGAGAGGAAGCCCUU[1]

Taft (FANTOM4) UCCUGCAGAGAGGAAGCCCUUC[1]

68 AAUAAAAAUUGAAUAGUGAGCAACCCAGUGGGCUAUGGAAAUGUGUGGAAGAUGGCAUUUCUAUUUCUCAGUGGGGCUCUUACCUAUUACUCAUCAAC

...........(((((((((...((((.((((..((((((((((..........))))))))))..)))).))))...)))).)))))..........

Morin (hEB) ACCCAGUGGGCUAUGGAAAUG[1]

Morin (hEB) UUUCUAUUUCUCAGUGGGGCUC[1]

Morin (hESC) AACCCAGUGGGCUAUGGA[1]

Morin (hESC) AACCCAGUGGGCUAUGGAAAUGU[1]

Morin (hESC) UUUCUAUUUCUCAGUGGGGCU[2]

Morin (hESC) AACCCAGUGGGCUAUGGAAAUG[2]

Morin (hESC) UUUCUAUUUCUCAGUGGGGCUC[2]

Seila (GSE13483) AACCCAGUGGGCUAUGGAAAUG[3]

Seila (GSE13483) UUUCUAUUUCUCAGUGGGGCUC[2]

69 CCCGGGACCUUGGUCCAGGCGCUGGUCUGCGUGGUGCUCGGGUGGAUAAGUCUGAUCUGAGCACCACACAGGCCGGGCGCCGGGACCAAGGGGGCUC

(((....(((((((((.((((((((((((.((((((((((((((((....))).))))))))))))).)))))).)))))).))))))))))))...

Morin (hEB) GCUGGUCUGCGUGGUGCUCGGGU[2]

Morin (hEB) UGAGCACCACACAGGCCGGGC[1]

Morin (hEB) UGAGCACCACACAGGCCGGGCG[2]

Morin (hEB) UGAGCACCACACAGGCCGG[2]

Morin (hEB) GCUGGUCUGCGUGGUGCUCGG[4]

Morin (hEB) UGAGCACCACACAGGCCGGG[1]

Morin (hEB) UGAGCACCACACAGGCCGGGCGC[4]

Morin (hESC) GCUGGUCUGCGUGGUGCUCGGGU[1]

70 AGUCAGAAUUCUCAUCAGGCUGUGAUGCUCAGUUGUGUGUAGAUUGAAAGCCCUAAUUUUACACACAACUGAGGAUCAUAGCCUGAUGGUUCCUUUUUGU

...(((((...(((((((((((((((.((((((((((((((((..............)))))))))))))))).))))))))))))))).....))))).

ENCODE (GSE14362) UGAUGCUCAGUUGUGUGUAGA[1]

ENCODE (GSE14362) UGUGAUGCUCAGUUGUGUGUAG[1]

ENCODE (GSE14362) UUACACACAACUGAGGAUCAU[1]

Morin (hEB) UGAUGCUCAGUUGUGUGU[1]

Morin (hEB) UUACACACAACUGAGGAUCAUA[3]

Morin (hEB) GUGAUGCUCAGUUGUGUGUAG[1]

Wyman (GSM379267) UUACACACAACUGAGGAUC[2]

71 CCCUCCACUCGGAGGCCAUCACUGGACUUGGAGUCAGAAGAGUGGAGUCGGGUCAGACUUCAACUCUGACUUUGAAGGUGGUGAGUGCCUCAAACUCAA

...........((((((((((((....(..((((((((....(((((((......)))))))..))))))))..).)))))))...)))))........

Zhu (GSE14738) ACUGGACUUGGAGUCAGAAG[1]

ENCODE (GSE14362) ACUGGACUUGGAGUCAGAA[9]

ENCODE (GSE14362) ACUGGACUUGGAGUCAGAAG[61]

ENCODE (GSE14362) ACUGGACUUGGAGUCAGAAGA[35]

ENCODE (GSE14362) CUGGACUUGGAGUCAGAAG[6]

Morin (hEB) ACUGGACUUGGAGUCAGAA[132]

Morin (hEB) CUGGACUUGGAGUCAGAAG[13]

Morin (hEB) ACUGGACUUGGAGUCAGAAGAG[2]

Morin (hEB) ACUGGACUUGGAGUCAGAAG[556]

Morin (hEB) CUGGACUUGGAGUCAGAA[4]

Morin (hEB) ACUGGACUUGGAGUCAGA[112]

Morin (hEB) UCACUGGACUUGGAGUCAGA[1]

Morin (hEB) ACUGGACUUGGAGUCAGAAGA[56]

Morin (hEB) CACUGGACUUGGAGUCAG[2]

Morin (hEB) CACUGGACUUGGAGUCAGAAG[2]

Morin (hESC) CUGGACUUGGAGUCAGAA[2]

Morin (hESC) ACUGGACUUGGAGUCAGAAG[401]

Morin (hESC) ACUGGACUUGGAGUCAGAAGAG[1]

Morin (hESC) CUGGACUUGGAGUCAGAAG[9]

Morin (hESC) ACUGGACUUGGAGUCAGAA[70]

Morin (hESC) ACUGGACUUGGAGUCAGA[172]

Morin (hESC) ACUGGACUUGGAGUCAGAAGA[17]

Morin (hESC) CACUGGACUUGGAGUCAG[1]

Morin (hESC) UCACUGGACUUGGAGUCAGAAG[1]

Taft (FANTOM4) ACUGGACUUGGAGUCAGA[71]

Taft (FANTOM4) ACUGGACUUGGAGUCAGAA[38]

Taft (FANTOM4) ACUGGACUUGGAGUCAGAAG[937]

Taft (FANTOM4) ACUGGACUUGGAGUCAGAAGA[35]

Taft (FANTOM4) CACUGGACUUGGAGUCAGAAG[5]

Taft (FANTOM4) CUGGACUUGGAGUCAGAA[6]

Taft (FANTOM4) CUGGACUUGGAGUCAGAAG[70]

Taft (FANTOM4) CUGGACUUGGAGUCAGAAGA[2]

Taft (FANTOM4) UGGACUUGGAGUCAGAAG[6]

Wyman (GSM379265) ACUGGACUUGGAGUCAGAA[1]

Wyman (GSM379265) CUGGACUUGGAGUCAGAAG[1]

Wyman (GSM379266) ACUGGACUUGGAGUCAGAA[3]

Wyman (GSM379267) ACUGGACUUGGAGUCAGAA[14]

Wyman (GSM379267) CUGGACUUGGAGUCAGAAG[3]

Wyman (GSM379268) ACUGGACUUGGAGUCAGAA[39]

Wyman (GSM379268) CUGGACUUGGAGUCAGAAG[11]

Wyman (GSM379268) CACUGGACUUGGAGUCAGA[1]

72 CACCCAGCAGGCGCAGGUCCUGUGCAGCAGGCCAACCGAGAAGCGCCUGCGUCUCCCAUUUUCGGGCUGGCCUGCUGCUCCGGACCUGUGCCUGAUCUUAA

.......(((((((((((((.(.(((((((((((.((((((((((....)))......)))))))..))))))))))).).))))))))))))).......

Zhu (GSE14738) UGUGCAGCAGGCCAACCGAG[1]

ENCODE (GSE14362) UGUGCAGCAGGCCAACCGAGA[1]

Morin (hEB) UUCGGGCUGGCCUGCUGCU[1]

Morin (hEB) GGGCUGGCCUGCUGCUCCG[1]

Morin (hEB) UUCGGGCUGGCCUGCUGCUCCGG[1]

Morin (hEB) GGGCUGGCCUGCUGCUCCGG[1]

Morin (hESC) CGGGCUGGCCUGCUGCUCCGG[1]

Morin (hESC) UUCGGGCUGGCCUGCUGCUCC[1]

Morin (hESC) UUCGGGCUGGCCUGCUGCUCCGG[3]

Morin (hESC) UUCGGGCUGGCCUGCUGCUCCGGA[1]

Seila (GSE13483) CGGGCUGGCCUGCUGCUCCGGA[1]

Seila (GSE13483) UCGGGCUGGCCUGCUGCUCCGG[1]

Seila (GSE13483) UCGGGCUGGCCUGCUGCUCCGGA[1]

Seila (GSE13483) UUCGGGCUGGCCUGCUGCUCCGG[3]

Taft (FANTOM4) UGUGCAGCAGGCCAACCGA[1]

Taft (FANTOM4) UGUGCAGCAGGCCAACCGAGA[2]

Taft (FANTOM4) UUCGGGCUGGCCUGCUGCUCCGG[1]

Taft (FANTOM4) UUCGGGCUGGCCUGCUGCUCCGGA[1]

73 UGCCCUGUUCUCCAUCCUGGGCCUCACCGGAGCUUUGCCACAGCAGCACUUGAGCCGGGGGCCAUAGUGCUUGUGGACAAGGACGGCGAGGCCAAGGAGAGCCAGCA

.((...(((((((......((((((.(((...((((((((((..((((((((.(((...))))).))))))))))).))))).))).))))))..)))))))..)).

Morin (hEB) UGCUUGUGGACAAGGACGGCGA[1]

Morin (hEB) UGCUUGUGGACAAGGACGG[1]

Morin (hEB) UGCUUGUGGACAAGGACGGCG[1]

Morin (hESC) UGCUUGUGGACAAGGACGGCGA[3]

Morin (hESC) UGCUUGUGGACAAGGACGG[1]

Morin (hESC) UGCUUGUGGACAAGGACGGCG[2]

74 UGAGAGUGUUGCCUUGAGGCCUAGAGCUCUGCUUUAUCUAAAUUUAAAAGGAGAUGGAAACUUAGAGAAGAGUAGAGGGUUCUGUAGUCUUGGGCAGCAAGCUCC

.(((..((((((((.(((((.((((((((((((((.(((((.(((..(......)..))).)))))..))))))))..))))))..)))))))))))))..))).

Morin (hEB) UUAGAGAAGAGUAGAGGGUUCUGU[3]

Morin (hESC) UUAGAGAAGAGUAGAGGGUUCUGU[3]

75 UAAUUUUGAAGCUGACUUUUUUAGGGAGUAGAAGGGUGGGGAGCAUGAACAAUGUUUCUCACUCCCUACCCCUCCACUCCCCAAAAAAGUCAGCUUCUCUUGUUA

.......(((((((((((((((.((((((.((.((((((((((...(((......)))...)))))))))).)).)))))).)))))))))))))))........

Morin (hESC) UUAGGGAGUAGAAGGGUGG[1]

Morin (hESC) UUAGGGAGUAGAAGGGUGGGG[2]

76 CAAGCCUUGGUUUCCUCAUCUAUAAAAUGAGGGCAGUAAGACCUUCCUUCCUUGUCUUACUACCCCCAUUUUAUAGAUGAGGAAACCAACCAGUG

......(((((((((((((((((((((((.(((.((((((((...........)))))))).))).)))))))))))))))))))))))......

Morin (hEB) UAUAAAAUGAGGGCAGUAAGAC[3]

Morin (hESC) UAUAAAAUGAGGGCAGUAAGAC[1]

77 AUCCACAGGUUCUUGGAAACUGUGACUUUAAGGGAAAUGGCGCACAGCAGACCCUGCAAUCAUGCCGUUUUGCUUGAAGUCGCAGUUUCCCAGGACCUCUCAC

......(((((((.((((((((((((((((((.(((((((((....((((...)))).....))))))))).)))))))))))))))))).))))))).....

ENCODE (GSE14362) UGUGACUUUAAGGGAAAUGG[1]

Morin (hEB) UGUGACUUUAAGGGAAAUGGCG[3]

Morin (hESC) UGUGACUUUAAGGGAAAUGGCG[15]

Morin (hESC) UGUGACUUUAAGGGAAAUG[1]

Wyman (GSM379266) CCGUUUUGCUUGAAGUCGC[1]

78 CUGUAAACUUGAAGGUAGGGAACUCUGUCUUCACUCAUGAGUACCUUCCAACACGAGCUCUCAGGAGUAAAGACAGAGUUCCCUACCUUCAAUGUGGAU

......(((((((((((((((((((((((((.((((.((((...(((.......)))..)))).)))).))))))))))))))))))))))).))....

ENCODE (GSE14362) AACUCUGUCUUCACUCAUGAGU[2]

ENCODE (GSE14362) AGGAGUAAAGACAGAGUUCC[1]

ENCODE (GSE14362) CAGGAGUAAAGACAGAGUU[13]

ENCODE (GSE14362) CAGGAGUAAAGACAGAGUUCC[2]

ENCODE (GSE14362) CUCAGGAGUAAAGACAGAGUU[1]

ENCODE (GSE14362) CUCUCAGGAGUAAAGACAGAGU[3]

ENCODE (GSE14362) UCAGGAGUAAAGACAGAGUU[7]

ENCODE (GSE14362) UCUCAGGAGUAAAGACAGAGU[4]

ENCODE (GSE14362) UCUCAGGAGUAAAGACAGAGUU[2]

ENCODE (GSE14362) UCUCAGGAGUAAAGACAGAGUUC[2]

Ender (GSM337570) UCAGGAGUAAAGACAGAGUUCC[1]

Morin (hEB) AACUCUGUCUUCACUCAUGAGU[2]

Morin (hEB) UCAGGAGUAAAGACAGAG[1]

Morin (hEB) UCAGGAGUAAAGACAGAGUUCC[5]

Morin (hEB) UCUCAGGAGUAAAGACAG[2]

Morin (hEB) UCAGGAGUAAAGACAGAGUUC[2]

Morin (hEB) CAGGAGUAAAGACAGAGUUCC[3]

Morin (hEB) CAGGAGUAAAGACAGAGUU[1]

Morin (hEB) UCUCAGGAGUAAAGACAGAGUU[12]

Morin (hEB) UCUCAGGAGUAAAGACAGAGU[4]

Morin (hEB) UCUCAGGAGUAAAGACAGA[1]

Morin (hEB) UCUCAGGAGUAAAGACAGAGUUC[6]

Morin (hESC) UCAGGAGUAAAGACAGAGUUCC[1]

Morin (hESC) CUCAGGAGUAAAGACAGAGU[1]

Morin (hESC) UCAGGAGUAAAGACAGAGUUC[1]

Morin (hESC) CAGGAGUAAAGACAGAGUUCC[1]

Morin (hESC) UCUCAGGAGUAAAGACAGAGUUCCC[1]

Morin (hESC) CAGGAGUAAAGACAGAGUUC[1]

Morin (hESC) UCUCAGGAGUAAAGACAGAGUU[8]

Morin (hESC) UCUCAGGAGUAAAGACAGAGU[2]

Morin (hESC) CAGGAGUAAAGACAGAGUUCCC[2]

Morin (hESC) UCUCAGGAGUAAAGACAGAGUUC[2]

79 GCGGCUGCUGCUCUCCGUUUAUCCCACCACUGCCACCAUUAUUGCUACUGUUCAGCAGGUGCUGCUGGUGGUGAUGGUGAUAGUCUGGUGGGGGCGGUGGGGCUGCUGUUGCUG

(((((.((.(((((((((...(((((((((((.(((((((((..(((.....((((....)))).)))..))))))))).))))..))))))))))).))))).)).)))))..

Morin (hEB) AUCCCACCACUGCCACCAUU[1]

Morin (hEB) AUCCCACCACUGCCACCAU[22]

Morin (hEB) AUCCCACCACUGCCACCA[129]

Morin (hESC) UAUCCCACCACUGCCACCA[1]

Morin (hESC) AUCCCACCACUGCCACCAUU[1]

Morin (hESC) GCGGCUGCUGCUCUCCGUUU[1]

Morin (hESC) AUCCCACCACUGCCACCAU[33]

Morin (hESC) AUCCCACCACUGCCACCA[274]

Seila (GSE13483) AUCCCACCACUGCCACCA[2]

Seila (GSE13483) AUCCCACCACUGCCACCAU[3]

Seila (GSE13483) UAUCCCACCACUGCCACCA[5]

Taft (FANTOM4) AUCCCACCACUGCCACCA[104]

Taft (FANTOM4) AUCCCACCACUGCCACCAU[3]

Taft (FANTOM4) AUCCCACCACUGCCACCAUU[1]

Taft (FANTOM4) UAUCCCACCACUGCCACCA[2]

Wyman (GSM379266) UAUCCCACCACUGCCACCA[1]

80 CAACAGCUUCAACAGCAAUUGGAGAAGAUUGCAGAGUAAGUUCCUGAUUAAGAAAUGGAAUUUACUCUGCAAUCUUCUCCAAUUGCUGUCACCAUCAUUC

...........(((((((((((((((((((((((((((((((((............)))))))))))))))))))))))))))))))))...........

Morin (hEB) GAAGAUUGCAGAGUAAGUUCC[1]

Morin (hEB) AAGAUUGCAGAGUAAGUUCCU[1]

Morin (hEB) AGAAGAUUGCAGAGUAAGUU[1]

Morin (hEB) AAUUUACUCUGCAAUCUUCUCC[1]

Morin (hESC) AGAAGAUUGCAGAGUAAGUUCC[3]

Morin (hESC) GAAGAUUGCAGAGUAAGUUCC[1]

81 CACUUUGGGGACUGGGGAUGAGGAGGCAUCUUGAGAAAUGGAAGGAAUGGGAUCUACUUCCAGUUCACUAGAGGCGUCCUGACACCCCUAGCUCAGCAUC

.......(((.((((((.((((((.((.(((.(.(((.((((((............)))))).))).).))).)).))))..)).)))))))))......

Morin (hEB) AGGAGGCAUCUUGAGAAAUGGA[5]

Morin (hEB) AGUUCACUAGAGGCGUCCUGAC[2]

Morin (hEB) AGUUCACUAGAGGCGUCCUGACA[3]

Morin (hEB) AGUUCACUAGAGGCGUCCU[1]

82 AACCAUUAGGGGGCUGUGGUUUGCCAGGGCAGGAGGUGGAAGGGAGCCCCAUUUACAGUGGUAACUUCCUUUCCCUUUCCAUCCUGGCAGGCUUCAGAGAACUUUACCAG

......(((((..(((.((((((((((((..(((((.(((((((((..((((.....))))....))))))))))))))..)))))))))))).)))....)))))....

Morin (hEB) UUUCCCUUUCCAUCCUGGCAG[1]

Morin (hESC) UUUCCCUUUCCAUCCUGGCAG[4]

83 GGUAAACCUUGGUGACUAAUUAGAGUCUGGCUGAUAUGGUUUGACACAGAGCUAAAUCAUAUGAACCAAACUCUAAUUAGUCAAUAAUUUCUGUU

((....))....((((((((((((((.(((.(.((((((((((.(.....).)))))))))).).))).))))))))))))))............

Morin (hEB) UAGAGUCUGGCUGAUAUGGUUU[7]

Morin (hEB) AAUCAUAUGAACCAAACUCUA[1]

Morin (hEB) UAGAGUCUGGCUGAUAUGG[1]

84 AUAUUUAUGCAUAUAUAUAGAGAUGUAUGGAAUCUGUAUAUAUCUAUAUAUAUGUGUAUAUAUAGAUUCCAUAAAUCUAUAUAUGUAUGUGUGUAUAUAU

((((.((((((((((((((.((((.((((((((((((((((((.(((....))).)))))))))))))))))).)))).)))))))))))))).))))..

Morin (hEB) AGAUGUAUGGAAUCUGUAUAUAUC[1]

Morin (hEB) AGAUGUAUGGAAUCUGUAUAUAUCU[2]

Morin (hEB) AGAUGUAUGGAAUCUGUAUAUAU[2]

Morin (hEB) AGAUGUAUGGAAUCUGUAUAUA[11]

Morin (hEB) AGAUGUAUGGAAUCUGUAUAU[7]

Morin (hESC) AGAUGUAUGGAAUCUGUAUAUAUC[1]

Morin (hESC) AGAUGUAUGGAAUCUGUAU[2]

Morin (hESC) AGAUGUAUGGAAUCUGUAUAUAU[2]

Morin (hESC) AGAUGUAUGGAAUCUGUAUAUA[14]

Morin (hESC) AGAUGUAUGGAAUCUGUAUAU[16]

85 CCAAAUGACUAGAGUAGACUUCAACUCCAAAUAAAAGUAAUUAAUUACAAAUGUAUUACAAAUAAAUUUACUUUUAUUUGGAGUCCAAGUCACUUCAGUCAUUUGGU

((((((((((..(((.(((((..(((((((((((((((((...........................)))))))))))))))))..))))))))..)))))))))).

Morin (hEB) CAACUCCAAAUAAAAGUA[9]

Morin (hEB) UCAACUCCAAAUAAAAGUA[3]

Morin (hESC) CAACUCCAAAUAAAAGUA[4]

Morin (hESC) UCAACUCCAAAUAAAAGUA[2]

Taft (FANTOM4) CAACUCCAAAUAAAAGUA[3]

86 CUUGCUGGGGCUGUGGAAAGAGCUCGUUGUUUGCUGGCCUGCUUUGUUGGUGACCAGCAUUGCACAUGGCUUUCUUUUCAGAUCAAACCCU

......(((..((((((((((((.((((((.((((((.(.(((.....)))).))))))..))).)))))..))))))))...))..))).

Morin (hEB) ACCAGCAUUGCACAUGGCU[3]

Morin (hESC) ACCAGCAUUGCACAUGGCU[6]

Morin (hESC) ACCAGCAUUGCACAUGGCUUU[1]

87 UUCAGUAUGACACCUCAAAGAAGCAAUACUGUUACCUGAAAUAGGCUGCGAAGAUAACAGUAUUUCAGAUAACAGUAUUACAUCUUUGAAGUGUCAUAUUCAC

...((((((((((.(((((((.(.((((((((((.(((((((..((((.........))))))))))).)))))))))).).))))))).))))))))))...

ENCODE (GSE14362) AAGCAAUACUGUUACCUGAAAU[1]

Morin (hEB) AAGCAAUACUGUUACCUGAAAU[2]

Morin (hEB) AGCAAUACUGUUACCUGAAAUA[1]

Morin (hEB) AGCAAUACUGUUACCUGAAA[1]

Morin (hESC) AAGCAAUACUGUUACCUGAA[1]

Morin (hESC) AAGCAAUACUGUUACCUGAAAU[4]

Taft (FANTOM4) UUUCAGAUAACAGUAUUACAU[1]

88 CACUGGAGUGGUCCCCAUUUUUCUCCCACUACCAGGCUCCCAUAAGGGUCGAAUGGGAUCCAGACAGUGGGAGAAAAAUGGGGACCACUAUCACCA

...(((((((((((((((((((((((((((..(.((.((((((.........)))))).)).)..)))))))))))))))))))))))).)))...

ENCODE (GSE14362) UGGGAUCCAGACAGUGGGAGA[1]

Morin (hEB) UGGGAUCCAGACAGUGGGAG[1]

Morin (hEB) UGGGAUCCAGACAGUGGGAGAA[1]

Morin (hESC) UGGGAUCCAGACAGUGGGAG[1]

Morin (hESC) UUCUCCCACUACCAGGCUCCC[2]

Morin (hESC) UUCUCCCACUACCAGGCUCCCA[1]

89 ACCAAUUCCUAGGUUGGUGCAAAAGUAAUUGCGGUCUUUGUCAUUAAAACCAAUAACAAAAACCACAAUUACUUUUUACUGACCUAAAGAUUAAUU

...((((..(((((..(((.(((((((((((.(((.(((((.(((......))).))))).))).))))))))))))))..)))))..))))....

ENCODE (GSE14362) AAAAACCACAAUUACUUUU[1]

Morin (hEB) AAAAGUAAUUGCGGUCUU[1]

Morin (hEB) CAAAAGUAAUUGCGGUCUUUGU[4]

Morin (hEB) AAAAGUAAUUGCGGUCUUUGU[5]

Morin (hEB) GCAAAAGUAAUUGCGGUCUUUGU[1]

Morin (hEB) CAAAAGUAAUUGCGGUCUUU[1]

Morin (hESC) CAAAAGUAAUUGCGGUCUUUGU[2]

Morin (hESC) AAAAGUAAUUGCGGUCUUUGUC[1]

Morin (hESC) GCAAAAGUAAUUGCGGUCUUUGUC[1]

Morin (hESC) AAAAGUAAUUGCGGUCUUUG[1]

Morin (hESC) AAAAGUAAUUGCGGUCUUUGU[6]

Morin (hESC) CAAAAGUAAUUGCGGUCUUUG[1]

Taft (FANTOM4) CAAAAGUAAUUGCGGUCUUUG[1]

Wyman (GSM379265) ACAAAAACCACAAUUACUU[3]

Wyman (GSM379265) CAAAAACCACAAUUACUUU[2]

Wyman (GSM379267) CAAAAACCACAAUUACUUU[2]

Wyman (GSM379267) ACAAAAACCACAAUUACUU[1]

Wyman (GSM379268) CAAAAACCACAAUUACUUU[2]

90 GACCAGAAGUGUUUUGGAUUUUGGACUUUUUCAGAUUUGGGGAUAUUUGCAUUAUACUUAUCCUAAAUCUGAAAGUCCAAAACCUGAAAUGACCAAUAAG

..........((((..(.((((((((((..((((((((((((..................)))))))))))))))))))))).)..))))..........

Morin (hEB) UUGGACUUUUUCAGAUUUGG[1]

Morin (hEB) UUGGACUUUUUCAGAUUUGGGGAU[2]

Morin (hEB) UUGGACUUUUUCAGAUUUG[1]

Morin (hEB) UUGGACUUUUUCAGAUUUGGGGAUA[1]

Morin (hEB) UGGACUUUUUCAGAUUUGGGGAU[1]

Morin (hEB) UUUGGACUUUUUCAGAUUUGG[1]

Morin (hEB) UGGACUUUUUCAGAUUUGGGGAUA[2]

Morin (hEB) UUGGACUUUUUCAGAUUUGGGGA[1]

Morin (hESC) UUUGGACUUUUUCAGAUUUGGG[1]

Morin (hESC) UUUGGACUUUUUCAGAUUUGGGGAU[1]

Morin (hESC) UUGGACUUUUUCAGAUUUGGGGAUAU[1]

Morin (hESC) UUUGGACUUUUUCAGAUUUG[1]

Morin (hESC) UUGGACUUUUUCAGAUUUGGGGAU[2]

Morin (hESC) UUGGACUUUUUCAGAUUUG[2]

Morin (hESC) UUGGACUUUUUCAGAUUUGGGGAUA[1]

Morin (hESC) UUUGGACUUUUUCAGAUUUGGGG[1]

Morin (hESC) UGGACUUUUUCAGAUUUG[1]

Morin (hESC) UGGACUUUUUCAGAUUUGGGG[1]

Morin (hESC) UGGACUUUUUCAGAUUUGGGGAUA[2]

Morin (hESC) UUGGACUUUUUCAGAUUUGGGGA[1]

91 CCAGCAUCAGCGUUACCUGGUAGUGAGUUAGAGAUGCAGAGCCCUGGGCUCCUCAGCAAACCUACUGGAUCUGCAUUUUAAUUCACAUGCAUGGUAAUGUCUGUAAAGCACU

...((..((((((((((..((((((((((.((((((((((.((.((((............))))..)).))))))))))))))))).)))..))))))).)))....))...

ENCODE (GSE14362) CCAGCAUCAGCGUUACCUGG[1]

ENCODE (GSE14362) UAGUGAGUUAGAGAUGCAGA[1]

ENCODE (GSE14362) UAGUGAGUUAGAGAUGCAGAG[5]

ENCODE (GSE14362) UAGUGAGUUAGAGAUGCAGAGC[1]

Morin (hEB) UAGUGAGUUAGAGAUGCAGAGC[4]

Morin (hEB) UAGUGAGUUAGAGAUGCAG[1]

Morin (hEB) UAGUGAGUUAGAGAUGCAGAGCCCU[2]

Morin (hEB) UAGUGAGUUAGAGAUGCAGAGCC[6]

Morin (hEB) UAGUGAGUUAGAGAUGCAGAGCCC[1]

Morin (hESC) UAGUGAGUUAGAGAUGCAGAGC[1]

Morin (hESC) UAGUGAGUUAGAGAUGCAGAG[1]

Morin (hESC) UAGUGAGUUAGAGAUGCAGAGCC[5]

Taft (FANTOM4) GAUCUGCAUUUUAAUUCACAU[1]

Taft (FANTOM4) UAGUGAGUUAGAGAUGCAGA[1]

92 CAGACGGCUCCCCUGGGGGGCGGGGAGAGAACGCAGUGACGUCUGGCCGCGUGCGCAUGUCGGGCGCUUUCUCCUCCCCCUACCCAGGGAGCCGCAC

..(.((((((((.(((((((.(((((((((.(((..((((((...((.....))..)))))).))).))))))))).)))..)))))))))))))..

Morin (hEB) CGGGGAGAGAACGCAGUGACGUC[1]

Morin (hESC) CGGGGAGAGAACGCAGUGACGU[2]

93 GUGUGCCGUCACCACGUGCCAUGUGUACACACGUGCCAGGCGCUGUCUUGAGACAUUCGCGCAGUGCACGGCACUGGGGACACGUGGCACUGGCUGAACUGCCAC

(((.((..((((((.(((((((((((...((.(((((..(((((((..((((...)))).)))))))..)))))))...)))))))))))))).)))...)))))

Morin (hEB) UGCACGGCACUGGGGACACGU[4]

Morin (hESC) UGCACGGCACUGGGGACACGUG[2]

Morin (hESC) GUGCACGGCACUGGGGACACGU[1]

Morin (hESC) UGCACGGCACUGGGGACACGU[3]

Taft (FANTOM4) UGUGUACACACGUGCCAGGCGCUG[2]

94 GCUUCAUGGAGGGCAUUAGGCAGUGGCCAGAGCCCUGCAGUGCUGGGCAUGGGCUUCUCGUGGGCUCUGGCCACGGCCCUGAGCUCCUCCCCUUAC

.......(((((((.((((((.(((((((((((((.((((.(((.......)))..)).))))))))))))))).).))))))).)))))......

ENCODE (GSE14362) CAGUGGCCAGAGCCCUGCA[1]

Morin (hEB) CUCGUGGGCUCUGGCCACGGC[2]

Morin (hEB) UCGUGGGCUCUGGCCACGGCC[1]

Morin (hEB) CUCGUGGGCUCUGGCCACGGCC[2]

Morin (hEB) UCGUGGGCUCUGGCCACGGC[2]

Morin (hESC) CUCGUGGGCUCUGGCCACGGC[3]

Morin (hESC) CUCGUGGGCUCUGGCCACGGCC[1]

Taft (FANTOM4) CAGUGGCCAGAGCCCUGCA[1]

Taft (FANTOM4) CAGUGGCCAGAGCCCUGCAG[27]

Taft (FANTOM4) CAGUGGCCAGAGCCCUGCAGU[1]

Taft (FANTOM4) CUCGUGGGCUCUGGCCACGG[1]

Taft (FANTOM4) CUCGUGGGCUCUGGCCACGGC[6]

Taft (FANTOM4) CUCGUGGGCUCUGGCCACGGCC[5]

95 GCAUGUUGAUUGCGAGUGUGUGGAGACAAAGGCAGUUCCCACCACAGUUAGGUCCUGGCCAUUGUUUCCUCGCCUGCGAUGCUCCUUGUA

(((.(..((((((.((.(((.((((((((.(((((..((...........))..)).))).)))))))).)))))))))).)..).))).

ENCODE (GSE14362) AAGGCAGUUCCCACCACAGUUAGGUC[1]

Morin (hEB) UGGAGACAAAGGCAGUUC[1]

Morin (hEB) UGGAGACAAAGGCAGUUCC[5]

Morin (hESC) UGGAGACAAAGGCAGUUCC[2]

96 AUUCAGAAUCCCAUCAAGCAGCAGCCAUUCAGUCUCUGUCUGUGGUUGGUUAAGUGGGACUGUGGAGACUGAAUGGCUGCUGCUUGAUUGGUUACAGAGU

((((.....((.(((((((((((((((((((((((((((((...............))))...))))))))))))))))))))))))).)).....))))

Morin (hEB) UGUGGAGACUGAAUGGCUG[2]

Morin (hESC) ACUGUGGAGACUGAAUGGCUG[1]

Morin (hESC) ACUGUGGAGACUGAAUGGCUGCUGCU[1]

Morin (hESC) UGUGGAGACUGAAUGGCUGCUG[1]

Morin (hESC) UGUGGAGACUGAAUGGCUGCU[2]

Morin (hESC) UGUGGAGACUGAAUGGCUG[1]

Morin (hESC) UGUGGAGACUGAAUGGCUGCUGC[1]

97 GAUCCAGGGAACCCUAGAGCAGGGGGAUGGCAGAGCAAAAUUCAUGGCCUACAGCUGCCUCUUGCCAAACUGCACUGGAUUUUGUGUCUCCCAUUCCCCAGAGCUGUCUGAGGUGCUUUG

....(((((.(((.((((((.(((((((((.(((((((((((((.(((........)))...(((......))).)))))))))).))).)))))))))...))..)))).))).)))))

ENCODE (GSE14362) AGGGGGAUGGCAGAGCAAAAUU[1]

Ender (GSM337571) UUUUGUGUCUCCCAUUCCCC[1]

Morin (hEB) AGGGGGAUGGCAGAGCAA[1]

Morin (hEB) UUUUGUGUCUCCCAUUCCCCAG[2]

Morin (hEB) AGGGGGAUGGCAGAGCAAAAUU[2]

Morin (hEB) AGGGGGAUGGCAGAGCAAA[2]

Morin (hEB) UUUUGUGUCUCCCAUUCCCC[2]

Morin (hESC) UUUUGUGUCUCCCAUUCCCCAG[5]

Morin (hESC) AGGGGGAUGGCAGAGCAAAAU[1]

Morin (hESC) CAGGGGGAUGGCAGAGCA[1]

Morin (hESC) AGGGGGAUGGCAGAGCAAAAUU[10]

Morin (hESC) CAGGGGGAUGGCAGAGCAAA[1]

Morin (hESC) AGGGGGAUGGCAGAGCAAAAUUC[1]

Seila (GSE13483) AGGGGGAUGGCAGAGCAA[3]

Taft (FANTOM4) AGGGGGAUGGCAGAGCAAAAU[1]

Taft (FANTOM4) AGGGGGAUGGCAGAGCAAAAUU[2]

Taft (FANTOM4) UUUUGUGUCUCCCAUUCCCCA[1]

Wyman (GSM379268) UUUUGUGUCUCCCAUUCCC[2]

98 AUUAGUCAACACAUGUGUACACAGCUCUGAUGAUCUCUAUGUAGAUGCCUAUAUAAAUAGCUAUAUAGAUAUCAUCAGAGCUGUGUAUAUAUGUGUUAGAGAUA

.....(((((((((((((((((((((((((((((.(((((((((....((((....))))))))))))).))))))))))))))))))))))))))).))....

Morin (hEB) AUAUAGAUAUCAUCAGAGCUGU[2]

Morin (hESC) AUAUAGAUAUCAUCAGAGCU[1]

99 AGAUGGUAUUGAGUGGAUGCUGUUAUAUAUACAGCCAUGCACUCUGUAGUUUGGGUACACAGUGCAUGGCUGUAUAUAUAACACUAUCCAUUCAUCUUUCAGC

...(((...((((((((((.(((((((((((((((((((((((.((((.......)))).))))))))))))))))))))))).))))))))))....)))..

Morin (hEB) UGCAUGGCUGUAUAUAUAACA[1]

Morin (hESC) GUGCAUGGCUGUAUAUAUAACA[3]

Morin (hESC) UUAUAUAUACAGCCAUGCACU[1]

Morin (hESC) UAUAUAUACAGCCAUGCACUC[1]

Morin (hESC) UUAUAUAUACAGCCAUGCACUCU[1]

100 CAGCGAGGGCGCGCUGGCCCUGGGCAGCGUGUGGCUGAAGGUCACCAUGUUCUCCUUGGCCAUGGGGCUGCGCGGGGCCAGCAGGUCCACGUCC

..(((.((((..((((((((((.(((((..((((((.((((............))))))))))...))))).))))))))))..)))).)))..

ENCODE (GSE14362) UUGGCCAUGGGGCUGCGCG[2]

ENCODE (GSE14362) UUGGCCAUGGGGCUGCGCGG[2]

Morin (hEB) UUGGCCAUGGGGCUGCGCGGG[1]

Morin (hESC) UUGGCCAUGGGGCUGCGCGG[2]

Morin (hESC) UUGGCCAUGGGGCUGCGCGGGGC[1]

Morin (hESC) UUGGCCAUGGGGCUGCGCGGG[1]

Morin (hESC) UUGGCCAUGGGGCUGCGCGGGG[1]

Seila (GSE13483) UUGGCCAUGGGGCUGCGCGGG[3]

Seila (GSE13483) UUGGCCAUGGGGCUGCGCGGGG[6]

Seila (GSE13483) UUGGCCAUGGGGCUGCGCGGGGC[4]

Taft (FANTOM4) CAGCGAGGGCGCGCUGGC[6]

Taft (FANTOM4) CAGCGAGGGCGCGCUGGCC[1]

Taft (FANTOM4) CCUGGGCAGCGUGUGGCUGAAGG[3]

Taft (FANTOM4) UUGGCCAUGGGGCUGCGCGGGGC[1]

101 UUAUCGAGGAAAAGAUCGAGGUGGGUUGGGGCGGGCUCUGGGGAUUUGGUCUCACAGCCCGGAUCCCAGCCCACUUACCUUGGUUACUCUCCUUC

.....(((((...((((((((((((((((((((((((.(((((......))))).))))))..)))))))))....)))))))))....))))).

Zhu (GSE14738) CAGCCCGGAUCCCAGCCCACUU[1]

Zhu (GSE14738) CAGCCCGGAUCCCAGCCCACUUAC[1]

ENCODE (GSE14362) CAGCCCGGAUCCCAGCCCACUU[1]

Morin (hEB) CAGCCCGGAUCCCAGCCCACU[1]

Morin (hEB) CAGCCCGGAUCCCAGCCCACUUA[1]

Morin (hESC) CAGCCCGGAUCCCAGCCCAC[1]

Morin (hESC) CAGCCCGGAUCCCAGCCCACUUAC[1]

Morin (hESC) CAGCCCGGAUCCCAGCCCACUUA[1]

Morin (hESC) GUGGGUUGGGGCGGGCUCU[1]

Morin (hESC) CAGCCCGGAUCCCAGCCC[1]

Morin (hESC) CAGCCCGGAUCCCAGCCCACUU[3]

102 AUAGAAAAUAAAACACAUACUGCUGUAUUGUCAGGUAGUGAUAGGAUUUAUCACUACCUGACAAUACAGUAUGUGUUUGUUUUAUAUAUUU

.......((((((((.((((((((((((((((((((((((((((...)))))))))))))))))))))))).)))).))))))))......

Morin (hEB) UGCUGUAUUGUCAGGUAGUGA[2]

Morin (hEB) AUUGUCAGGUAGUGAUAGG[1]

Morin (hESC) UGUAUUGUCAGGUAGUGAUAGG[1]

Morin (hESC) CUGCUGUAUUGUCAGGUAGUG[1]

Taft (FANTOM4) CUGCUGUAUUGUCAGGUAGUGA[1]

Taft (FANTOM4) CUGUAUUGUCAGGUAGUGA[1]

Taft (FANTOM4) UGCUGUAUUGUCAGGUAGUGA[1]

Wyman (GSM379267) UGCUGUAUUGUCAGGUAGU[1]

Wyman (GSM379268) UCACUACCUGACAAUACAG[1]

Wyman (GSM379268) ACUACCUGACAAUACAGUA[1]

103 GACCACCCCCGGGGUCACCUCUCUGGCCGUCUACCUUCCACACUGACAAGGGCCGUGGGGACGUAGCUGGCCAGACAGGUGACCCCAGAGCGGCGU

...(.((((.((((((((((.((((((((.((((.((((.(((.(.......).))))))).)))).)))))))).)))))))))).).).)).).

Morin (hEB) UGGGGACGUAGCUGGCCAGAC[1]

Morin (hESC) CGGGGUCACCUCUCUGGCCGUC[1]

Morin (hESC) UGGGGACGUAGCUGGCCAGACAG[1]

Morin (hESC) CUGACAAGGGCCGUGGGGACGUA[1]

Morin (hESC) UGGGGACGUAGCUGGCCAG[3]

Morin (hESC) UGGGGACGUAGCUGGCCAGAC[3]

Morin (hESC) UGGGGACGUAGCUGGCCAGACA[2]

Taft (FANTOM4) CUGGCCAGACAGGUGACCCCAGA[1]

104 CCUCUGUUCCUGAGGGUAGGAGGAUUCCUGUCCCCUUCAUACCAUCCUUGGUACUGAGAGCGAUGCUGAAGAGACUGGGAUGCUCAUAUUCUCACAAAGGGACU

.....(((((((((((((.(((.(((((.(((..(((((...(((((((........))).)))).))))).))).))))).))).)))))))....)))))).

Morin (hEB) AUGCUGAAGAGACUGGGAUGCU[1]

Morin (hEB) UGCUGAAGAGACUGGGAUGCU[2]

Morin (hESC) UGCUGAAGAGACUGGGAUG[1]

105 CUAUGAAAGCCAGAUUCAGCUUUCCCUUCAGAGCCUGGCUUUGGCAUCUAUGAAAGCCAGGCUCUGAAGGGAAAGUUGAAUCUUGCUAGAGUGG

((((...(((.(((((((((((((((((((((((((((((((.(......).))))))))))))))))))))))))))))))).)))...))))

ENCODE (GSE14362) UUUCCCUUCAGAGCCUGGCUUU[1]

Morin (hEB) AGCCAGGCUCUGAAGGGAAA[1]

Morin (hEB) UUUCCCUUCAGAGCCUGGCUUU[4]

Morin (hEB) UUUCCCUUCAGAGCCUGGCUU[1]

Morin (hESC) CUUUCCCUUCAGAGCCUGGCUUU[1]

Morin (hESC) UUUCCCUUCAGAGCCUGGCUUU[1]

Morin (hESC) CUUUCCCUUCAGAGCCUGGC[1]

106 CAAAGAAGGAGGUGGUCGAGGGAAUCUGAGAAGGCGCACAAGGUUUGUGUCCAAUACAGUCCACACCUUGCGCUACUCAGGUCUGCUCGUGCCCUCCCAUGAUA

.......(((((.(..((((.(.(((((((..((((((..((((..(((..(......)..))))))))))))).))))))).).))))..)))))).......

ENCODE (GSE14362) AAUCUGAGAAGGCGCACAAGGUUUGU[1]

ENCODE (GSE14362) GAAUCUGAGAAGGCGCACAAGGUUUG[2]

Morin (hEB) AAUCUGAGAAGGCGCACAAGGUUUG[2]

Morin (hEB) AAUCUGAGAAGGCGCACAAGGUUU[5]

Morin (hEB) CACCUUGCGCUACUCAGGUCUGCUCGU[8]

Morin (hEB) ACCUUGCGCUACUCAGGUCUGCUCGU[1]

Morin (hESC) AAUCUGAGAAGGCGCACAAGGUUUGU[1]

Morin (hESC) AAUCUGAGAAGGCGCACAAGGUUU[3]

Morin (hESC) CACCUUGCGCUACUCAGGUCUG[1]

Morin (hESC) AAUCUGAGAAGGCGCACAAGGUUUG[3]

Morin (hESC) CACCUUGCGCUACUCAGGUCUGCUCGU[6]

Morin (hESC) CACCUUGCGCUACUCAGGUCU[4]

Morin (hESC) GAAUCUGAGAAGGCGCACAAGGU[1]

Taft (FANTOM4) AAUCUGAGAAGGCGCACAAGGUU[1]

Taft (FANTOM4) CACCUUGCGCUACUCAGGU[1]

Taft (FANTOM4) CACCUUGCGCUACUCAGGUC[1]

Taft (FANTOM4) CACCUUGCGCUACUCAGGUCU[3]

Taft (FANTOM4) CACCUUGCGCUACUCAGGUCUG[2]

Taft (FANTOM4) CACCUUGCGCUACUCAGGUCUGC[4]

Wyman (GSM379267) CACCUUGCGCUACUCAGGU[1]

107 GCGCUGUCCUUCCUCUGGGGAGCAGGCUCCGGGGGACAGGGAAAAGCACACAAGGAACUUGUCCUCUAGGGCCUGCAGUCUCAUGGGAGAGUGACAUGCA

(((.((((((((((.(((((.((((((((..(((((((((.................)))))))))..))))))))..))))).)))).)).))))))).

ENCODE (GSE14362) UGUCCUCUAGGGCCUGCAGU[2]

ENCODE (GSE14362) UGUCCUCUAGGGCCUGCAGUCU[1]

Morin (hEB) UGUCCUCUAGGGCCUGCAGUC[1]

Morin (hEB) UGUCCUCUAGGGCCUGCAGUCU[6]

Morin (hESC) GUCCUCUAGGGCCUGCAGUCU[1]

Morin (hESC) UGUCCUCUAGGGCCUGCAGUC[1]

Morin (hESC) UGUCCUCUAGGGCCUGCAGUCU[5]

Taft (FANTOM4) UGUCCUCUAGGGCCUGCAGUC[2]

Taft (FANTOM4) UGUCCUCUAGGGCCUGCAGUCU[13]

Wyman (GSM379267) UGUCCUCUAGGGCCUGCAG[1]

108 GAGAGAAGAAUGCCCAACCAGCCCUCAGUUGCUACAGUUCCCUGUUGUUUCAGCUCGACAACAACAGGCGGCUGUAGCAAUGGGGGGCUGGAUGGGCAUCUCAA

..((((....((((((.(((((((((.(((((((((((..(((((((((((.....)).)))))))))..))))))))))).))))))))).))))))))))..

Morin (hEB) ACAGGCGGCUGUAGCAAUGG[1]

Morin (hEB) ACAGGCGGCUGUAGCAAUGGGG[3]

Morin (hEB) ACAGGCGGCUGUAGCAAUGGGGG[4]

Morin (hEB) CAACAGGCGGCUGUAGCAAUGGGG[1]

Morin (hEB) ACAGGCGGCUGUAGCAAU[2]

Morin (hEB) CAACAGGCGGCUGUAGCAA[1]

Morin (hESC) CAGUUGCUACAGUUCCCUGUUG[1]

Morin (hESC) CAACAGGCGGCUGUAGCAAU[1]

Morin (hESC) CAACAGGCGGCUGUAGCAAUGGGG[1]

Morin (hESC) ACAGGCGGCUGUAGCAAUGGGGG[7]

Morin (hESC) ACAGGCGGCUGUAGCAAUGGGG[5]

Morin (hESC) CAACAGGCGGCUGUAGCAAUGGGGGG[1]

Morin (hESC) CAGGCGGCUGUAGCAAUGGGG[2]

Morin (hESC) ACAGGCGGCUGUAGCAAU[3]

109 AGAUUCUCACCUGAACGCAUGACUCUUCAACCUCAGGACUUGCAGAAUUAAUGGAAUGCUGUCCUAAGGUUGUUGAGUUGUGCAUUUCUGGGCAUUUCA

.((......((.(((.(((..((((..((((((.(((((..(((............))).))))).))))))..))))..)))..))).)).....)).

ENCODE (GSE14362) CUGUCCUAAGGUUGUUGAGU[10]

ENCODE (GSE14362) CUGUCCUAAGGUUGUUGAGUU[24]

ENCODE (GSE14362) CUGUCCUAAGGUUGUUGAGUUG[2]

ENCODE (GSE14362) CUGUCCUAAGGUUGUUGAGUUGU[5]

ENCODE (GSE14362) UGUCCUAAGGUUGUUGAGUU[2]

ENCODE (GSE14362) UGUCCUAAGGUUGUUGAGUUG[1]

Ender (GSM337570) CUGUCCUAAGGUUGUUGAGUU[1]

Morin (hEB) UCUUCAACCUCAGGACUUGCA[13]

Morin (hEB) UCUUCAACCUCAGGACUU[1]

Morin (hEB) CUGUCCUAAGGUUGUUGAGUUGU[4]

Morin (hEB) CUGUCCUAAGGUUGUUGAGUUG[1]

Morin (hEB) UCUUCAACCUCAGGACUUGC[1]

Morin (hEB) CUGUCCUAAGGUUGUUGAGUU[15]

Morin (hEB) ACUCUUCAACCUCAGGACUUGCA[1]

Morin (hESC) UCUUCAACCUCAGGACUUGCA[6]

Morin (hESC) UGUCCUAAGGUUGUUGAGUU[2]

Morin (hESC) UCUUCAACCUCAGGACUUGCAG[1]

Morin (hESC) CUCUUCAACCUCAGGACUUGCA[1]

Morin (hESC) CUGUCCUAAGGUUGUUGAGUUGU[4]

Morin (hESC) CUGUCCUAAGGUUGUUGA[1]

Morin (hESC) CUUCAACCUCAGGACUUGCA[1]

Morin (hESC) UCUUCAACCUCAGGACUUGC[1]

Morin (hESC) CUGUCCUAAGGUUGUUGAGUU[28]

Wyman (GSM379267) CUGUCCUAAGGUUGUUGAG[1]

110 CAGAUGCCUAUAAUACGUCCAAUCUCAUGACACUCACUCACUAUCAUGAGAACAGCAUAGUGAGUGAGUCUCAUGAGAUCUGAUAUAUUAUAGGGGAAA

....(.(((((((((..((..(((((((((.(((((((((((((..((....))..))))))))))))).)))))))))..))..))))))))).)...

Morin (hEB) AAUCUCAUGACACUCACUCAC[1]

Morin (hEB) AAUCUCAUGACACUCACUCACU[3]

Morin (hEB) UGAGUGAGUCUCAUGAGAUCUGAU[1]

Morin (hESC) AAUCUCAUGACACUCACUCACU[3]

Morin (hESC) AAUCUCAUGACACUCACUCAC[1]

111 GGACUUGGCUCUAGGAUGUGCUCAUUGCAUGGGCUGUGUAUAGUAUUAUUCAAUACCCAGAGCAUGCAGUGUGAACAUAAUAGAGAUUGAAAGA

...(((..(((((..((((.(.(((((((((..(((.((((.(.......).)))).)))..))))))))).).))))..))))).....))).

Morin (hEB) UCAUUGCAUGGGCUGUGUAUAG[1]

Morin (hEB) UACCCAGAGCAUGCAGUGUGAAC[1]

Morin (hEB) CUCAUUGCAUGGGCUGUGU[1]

Morin (hEB) CUCAUUGCAUGGGCUGUGUAUA[5]

Morin (hEB) UACCCAGAGCAUGCAGUGUGAACA[1]

Morin (hEB) CUCAUUGCAUGGGCUGUGUAUAGUA[1]

Morin (hEB) UACCCAGAGCAUGCAGUGUGAA[8]

Morin (hEB) UACCCAGAGCAUGCAGUGUGA[1]

Morin (hEB) CUCAUUGCAUGGGCUGUGUAUAGU[1]

Morin (hEB) UGCUCAUUGCAUGGGCUGUGU[1]

Morin (hEB) CCCAGAGCAUGCAGUGUGAA[1]

Morin (hEB) UACCCAGAGCAUGCAGUGUG[1]

Morin (hESC) CUCAUUGCAUGGGCUGUGUAU[9]

Morin (hESC) UCAUUGCAUGGGCUGUGUAUAG[1]

Morin (hESC) UCAUUGCAUGGGCUGUGUAUA[1]

Morin (hESC) CUCAUUGCAUGGGCUGUGU[5]

Morin (hESC) CUCAUUGCAUGGGCUGUGUAUA[7]

Morin (hESC) UACCCAGAGCAUGCAGUGUGAA[15]

Morin (hESC) CCCAGAGCAUGCAGUGUGAACA[1]

Morin (hESC) UACCCAGAGCAUGCAGUGUGA[3]

Morin (hESC) UCAUUGCAUGGGCUGUGUAUAGU[1]

Morin (hESC) UACCCAGAGCAUGCAGUGUG[5]

112 AUCAUUUGCUCGGCAUCUGCUGAGUACCGCCAUGUCUGUUGGGCAUCCACAGUCUCCCACCAGGCAUUGUGGUCUCCGCUGACGCUUUGCCAUUUC

.......((..(((.((.((.(((.(((((.(((((((.((((...(....)...)))).))))))).)))))))).)).)).)))..))......

Morin (hEB) UGAGUACCGCCAUGUCUGUUGGG[6]

Morin (hEB) UGAGUACCGCCAUGUCUGUU[4]

Morin (hEB) UGAGUACCGCCAUGUCUGUUG[3]

Morin (hEB) UGAGUACCGCCAUGUCUGUUGG[1]

Morin (hESC) UGAGUACCGCCAUGUCUGUU[3]

Morin (hESC) UGAGUACCGCCAUGUCUGUUGGG[3]

Morin (hESC) CACCAGGCAUUGUGGUCUC[1]

Morin (hESC) UGAGUACCGCCAUGUCUGUUGG[3]

Morin (hESC) GAGUACCGCCAUGUCUGUUGG[1]
